# Undiagnosed G6PD deficiency in Black and Asian individuals is prevalent and contributes to health inequalities in type 2 diabetes diagnosis and complications

**DOI:** 10.2337/dc25-0556

**Published:** 2025-11-01

**Authors:** Susan Martin, Miriam Samuel, Daniel Stow, Alys M. Ridsdale, Ji Chen, Katherine G. Young, Harry D. Green, Genes & Health Research Team, Eamonn Maher, Shabana Chaudhary, Joseph Gafton, Karen A Hunt, Shapna Hussain, Kamrul Islam, Hilary Martin, Mohammed Bodrul Mazid, Elizabeth Owor, Jessry Russell, Nishat Safa, John Solly, Marie Spreckley, David A Van Heel, Jan Whalley, Ishevanhu Zengeya, Emily Mantle, Shaheen Akhtar, Samina Ashraf, Dan Mason, John Wright, Daniel MacArthur, Michael Simpson, Richard C Trembath, Gerome Breen, Raymond Chung, Sang Hyuck Lee, Omar Asgar, Joanne Harvey, Karen Tricker, Caroline Winckley, Hanifa Khatun, Amna Asif, Claudia Langenberg, Grainne Colligan, Ceri Durham, Bill Newman, Ahsan Khan, Teng Heng, Matt Hurles, Vivek Iyer, Georgios Kalantzis, Vladimir Ovchinnikov, Iaroslav Popov, Klaudia Walter, Panos Deloukas, David Collier, Ana Angel, Saeed Bidi, Fabiola Eto, Sarah Finer, Chris Griffiths, Sam Hodgson, Benjamin M Jacobs, Rohini Mathur, Caroline Morton, Asma Qureshi, Stuart Rison, Annum Salman, Miriam Samuel, Moneeza K Siddiqui, Daniel Stow, Sabina Yasmin, Julia Zöllner, Sheik Dowlut, Andrew T. Hattersley, Veline L’Esperance, Trevelyan J. McKinley, Sarah Finer, Inês Barroso

**Affiliations:** 1 Exeter Centre of Excellence for Diabetes Research (EXCEED), University of Exeter Medical School, Royal Devon & Exeter Hospital, Exeter, UK; 2 Wolfson Institute of Population Health, Queen Mary University of London, London, UK

**Keywords:** G6PD deficiency, type 2 diabetes, HbA1c, health inequalities

## Abstract

**Objective:**

Glucose-6-phosphate dehydrogenase (G6PD) deficiency presents silently and is not routinely screened. It is associated with markedly lower HbA1c for the prevailing glucose levels. Since HbA1c is internationally recommended to diagnose and manage type 2 diabetes (T2D), we investigated the population-level impact of undiagnosed G6PD deficiency on T2D diagnosis and complications in the UK.

**Research Design and Methods:**

We used whole-exome sequencing and electronic health record data from UK Biobank (N=467,368) and Genes & Health (N=43,011) cohorts.

**Results:**

In the UK, we estimated that approximately 1 in 7 Black and 1 in 63 Asian males carry G6PD deficiency alleles, compared to fewer than 1 in 10,000 White males. Despite this, less than 1 in 50 G6PD deficient men are clinically recognised. Male *G6PD* carriers have considerably lower average HbA1c (0.9% [IFCC: 10.0mmol/mol]) compared to non-carriers, while differences in average glucose were negligible. G6PD deficient men had 1.37 [95% CI: 1.01, 1.86] higher odds of developing diabetes-related microvascular complications than non-carriers. Although risk factors were similar prior to diagnosis, male *G6PD* carriers diagnosed with T2D since 2011 were on average 4.1 years [95% CI: 0.6, 7.7] older at diagnosis compared to non-carriers. In addition, lower mean HbA1c values in *G6PD* carriers falsely under-estimated their 10-year T2D risk.

**Conclusions:**

Undiagnosed G6PD deficiency has significant impact on T2D diagnosis with HbA1c and associates with increased risk of diabetes complications. This has major implications on global populations using HbA1c for diagnosis and monitoring, and could contribute significantly to inequalities in diabetes outcomes.

## Introduction

Glucose-6-phosphate dehydrogenase (G6PD) deficiency is an enzymatic disorder caused by mutations in the *G6PD* gene on the X chromosome(1; 2). It is estimated to affect over 400 million individuals, or 7.5% of the world population (3). Due to its X-linkage it is more common in men(4), but a large proportion of G6PD deficiency cases(5) are undiagnosed as the disease remains clinically silent. The majority of those affected descend from Africa, Asia, the Middle East and Mediterranean regions, due to its evolutionary advantage in conferring protection against severe malaria(6). Despite being so common and the World Health Organisation (WHO) recommending routine testing for G6PD deficiency in populations with over 5% prevalence(7), the condition is still not routinely screened for in most countries(8).

Previous studies have found that specific variants in the *G6PD* gene are common in African American (Asahi rs1050828 variant; minor allele frequency (MAF) = 11.6%)(9) and South Asian populations (Mediterranean rs5030868 variant; MAF = 1.7% in South Asia)(10). These *G6PD* variants are associated with lower glycated haemoglobin (HbA1c) for any given glucose level, particularly in males, a result of the deficient G6PD protein and consequent shortened lifespan of red blood cells(9; 11). The Asahi rs1050828 variant has been associated with delays in the treatment of type 2 diabetes (T2D) and increased risk of diabetes-associated complications(12; 13). These latter associations may result from the use of HbA1c to monitor treatment response or from the use of HbA1c to diagnose T2D, as it has been implemented in many countries based on WHO guidance in 2011(14; 15). Since G6PD deficiency normally presents silently and is not routinely screened, the population-level impact of undiagnosed G6PD deficiency on T2D diagnosis is unknown. Previous case-control studies did not estimate the prevalence of undiagnosed G6PD deficiency in a diverse population like the UK or estimate the impact of *G6PD* carrier status on delays-in-T2D diagnosis. In addition, the impact of the Mediterranean rs5030868 variant on diabetes diagnosis and diabetes-related complications in Asians has not been previously studied.

We therefore sought to evaluate the prevalence of undiagnosed G6PD deficiency in two UK population-based cohorts with availability of genetic and longitudinal health data, and assess its impact on T2D diagnosis and progression to complications. Our findings have major global significance(16), particularly in African ancestry populations that have implemented WHO guidance supporting the use of HbA1c to diagnose T2D.

## Research Design and Methods

### UK Biobank

The UK Biobank resource recruited volunteers aged 40–75 years from across the UK between 2006 and 2010(17). The UK Biobank has approval from the North West Multi-centre Research Ethics Committee (MREC) (http://www.ukbiobank.ac.uk/ethics/), and these ethical regulations cover the work in this study. Written informed consent was obtained from all participants. Whole-exome sequencing (WES) data, assessment centre interview data and linked electronic health record (EHR) data was available for 467,368 participants.

### Genes & Health

The Genes & Health study recruited UK volunteers aged over 16 years, identifying as Bangladeshi or Pakistani, and has sites in London, Bradford, and Manchester(18). Ethical approval for the study was provided by the South East London National Research Ethics Committee (14/LO/1240) with consent for publishing (http://www.genesandhealth.org/volunteer-information). Volunteers have consented to lifelong EHR linkage, DNA extraction and genomic analyses, as well as invitation to recall studies. WES data and linked EHR data was available for 43,011 participants.

Details of WES for both cohorts are given in the Supplemental Material.

### Biomarkers

We used HbA1c and random glucose measures collected at the baseline assessment centre for the UK Biobank, and those obtained in primary care for Genes & Health. As HbA1c measures from the UK Biobank assessment centre are systematically lower compared to measures obtained in primary care within a 100-day window, we applied a HbA1c recalibration (details in the Supplemental Material). However, as in our analyses we are comparing HbA1c measures between participants with different *G6PD* genotypes, the absolute measures of HbA1c are not important and the recalibration does not materially affect our results or their interpretation.

UK Biobank used a validated approach for all sample collection procedures(17). At baseline, all participants donated a non-fasted peripheral venous blood sample. Collecting and processing fasted blood samples in this very large population study with distributed assessment centres was logistically too challenging so non-fasted samples were collected. For Genes & Health, HbA1c and plasma glucose values were extracted from routine clinical care delivered by the UK’s National Health Service (NHS), and are deemed high enough quality with which to make clinical decisions and diagnoses, via established Genes & Health pipelines(18). Further details of biomarker data collection are given in the Supplemental Material.

### Clinical codes

Clinical characteristics of the study population were defined using primary and secondary care EHR data in addition to self-reported data where applicable. For the UK Biobank, EHR data was curated from assessment centre interview (self-reported), primary care (read v2 and v3 coded; which are currently only available for half the cohort) and secondary care (International Classification of Diseases (ICD)-9, ICD-10 and Office of Population Censuses and Surveys Classification of Interventions and Procedures (OPCS)-4 coded) sources. When dates of records were required, ICD-9 codes could not be used as associated dates were not available. For Genes & Health, EHR data was curated from primary care (Systematized Nomenclature of Medicine (SNOMED) coded) and secondary care (ICD-10 and OPCS-4 coded) sources. Information on clinical phenotype definitions is given in the Supplemental Material and code lists used to define these are available for re-use and review at: https://github.com/susiemartin/G6PD_deficiency.

### Statistical analysis

An analysis plan is given in Figure S1. Where we analysed the same genetic variant in both UK Biobank and Genes & Health cohorts, we conducted analyses for each cohort before applying a meta-analysis. This allowed us to adjust for the differences in ethnic composition (UK Biobank Asians are predominantly of Indian descent, whereas Genes & Health consists only of Bangladeshi and Pakistani individuals) and outcome definitions across cohorts.

### G6PD deficiency prevalence

For the UK Biobank and Genes & Health cohorts, WES data was extracted for the Asahi G6PD deficiency rs1050828-T and Mediterranean G6PD deficiency rs5030868-A alleles, and used to calculate the prevalence of each G6PD deficiency allele across sexes and self-reported ethnicity groups. We calculated the proportion of individuals with a G6PD deficiency diagnosis in carriers and non-carriers of G6PD deficiency alleles using EHR.

### HbA1c and random glucose levels

To compare HbA1c and random glucose across carrier status of G6PD deficiency alleles, we used statistical tests and effect size estimates chosen based on the number of genotypes being compared and whether parametric assumptions were met. We excluded individuals with conditions known to affect HbA1c readings(19–22), specifically those with a diagnosis of diabetes (any type) or pregnant at time of measurement. In a separate analysis, we also compared HbA1c and random glucose across carrier status in individuals with a prevalent diagnosis of diabetes (any type) at time of measurement.

### Diabetes-related complications

We compared the presence of diabetes-related microvascular and macrovascular complications across carrier status of G6PD deficiency alleles using logistic regression models. Meta-analysis across ethnic groups and cohorts was conducted for regression model analyses.

### Age of T2D diagnosis

Age of T2D diagnosis was defined as the earliest age at which a diagnosis of T2D was first recorded. In 2011, the WHO suggested HbA1c could be used to diagnose diabetes(14). Since then, it has become the routine test to diagnose diabetes in the UK, with 50 million tests being conducted annually, and guidelines recommending that HbA1c should not be used for diagnosis in individuals with haemolysis(23–25). Since we wanted to investigate the effect of *G6PD* carrier status on T2D diagnosis, we excluded those diagnosed before 2011. For those diagnosed since 2011, we then compared age of T2D diagnosis, across carrier status of G6PD deficiency alleles using linear regression models, as well as appropriate statistical tests and effect size estimates.

### T2D risk prediction in simulated individuals

QDiabetes-2018 prediction models estimate the 10-year risk of developing T2D(26), and are recommended for diabetes risk assessment in the NHS Health Check(27). QDiabetes-2018 models are incorporated into the EHR software used by 58% of general practitioners (GPs) in England(26), and National Institute for Health and Care Excellence (NICE) guidelines in the UK recommend that individuals with calculated high risk scores would be offered HbA1c or fasting glucose testing(23). NICE Clinical Knowledge Summaries (CKS), used by GPs, do not recommend using both tests and in fact recommend confirming diabetes diagnosis with the same test(19; 20). QDiabetes-2018 score A incorporates clinical risk factors, and if an individual is classed as high-risk (10-year risk >= 5.6%), then guidelines recommend a HbA1c test is performed and the more detailed score C, which also includes HbA1c and has differing high-risk threshold, is then calculated. To determine how HbA1c levels affect T2D risk prediction, we simulated two individuals with identical clinical risk factors based on the averages for male UK Biobank participants of Black ethnicity with high-risk score A and subsequent diagnosis of T2D. We gave these individuals differing HbA1c levels to reflect the average HbA1c of carriers and non-carriers of G6PD deficiency alleles in this cohort, and calculated and compared their respective QDiabetes-2018 scores A and C.

### T2D risk factors in real-world individuals

To compare the clinical risk factors of real-world *G6PD* carriers and non-carriers that did not have T2D at baseline, we used UK Biobank and Genes & Health participants with subsequent diagnosis of T2D, calculating QDiabetes-2018 scores A and C for these individuals. For male UK Biobank participants of Black ethnicity with high-risk score A (>=5.6%) and subsequent diagnosis of T2D, carriers and non-carriers of G6PD deficiency alleles were compared in groups with matching characteristics. We also compared HbA1c levels in participants with characteristics matching each of the above simulated individuals. Matching was conducted using (a) clinical risk factors and (b) score A, and score C was compared across the matched groups of carriers and non-carriers.

### Net reclassification index

To assess the ability of QDiabetes-2018 scores A and C to predict future T2D diagnosis, we compared their prediction performance using categorical net reclassification index (NRI) in UK Biobank participants. Details of the NRI calculation are given in the Supplemental Material. Cases were defined as those with incident T2D diagnoses after baseline visit, and non-cases were defined as those without T2D diagnoses at any time. Confidence intervals for NRI were generated using bootstrapping with 1,000 iterations.

Summary cohort characteristics are given in Table S1, and additional information on statistical analyses is given in the Supplemental Material.

## Results

In UK Biobank, we found that 15.1% (1 in 7) of males of self-reported Black ethnicity carried the Asahi G6PD deficiency rs1050828-T allele, compared to <0.1% of self-reported White and Asian individuals (Table 1). The Mediterranean G6PD deficiency allele (rs5030868-A) was found in 1.6% (UK Biobank) and 1.3% (Genes & Health) of Asian males (approximately 1 in 63) and was equally rare in other ethnic groups (Table 1). Only <1.1% of Black and <2.9% of Asian males with a G6PD deficiency allele had a diagnosis of G6PD deficiency in their health records (Table 1), highlighting the under-diagnosis of G6PD deficiency. Results for women are given in Table S2.

**Figure 1:**
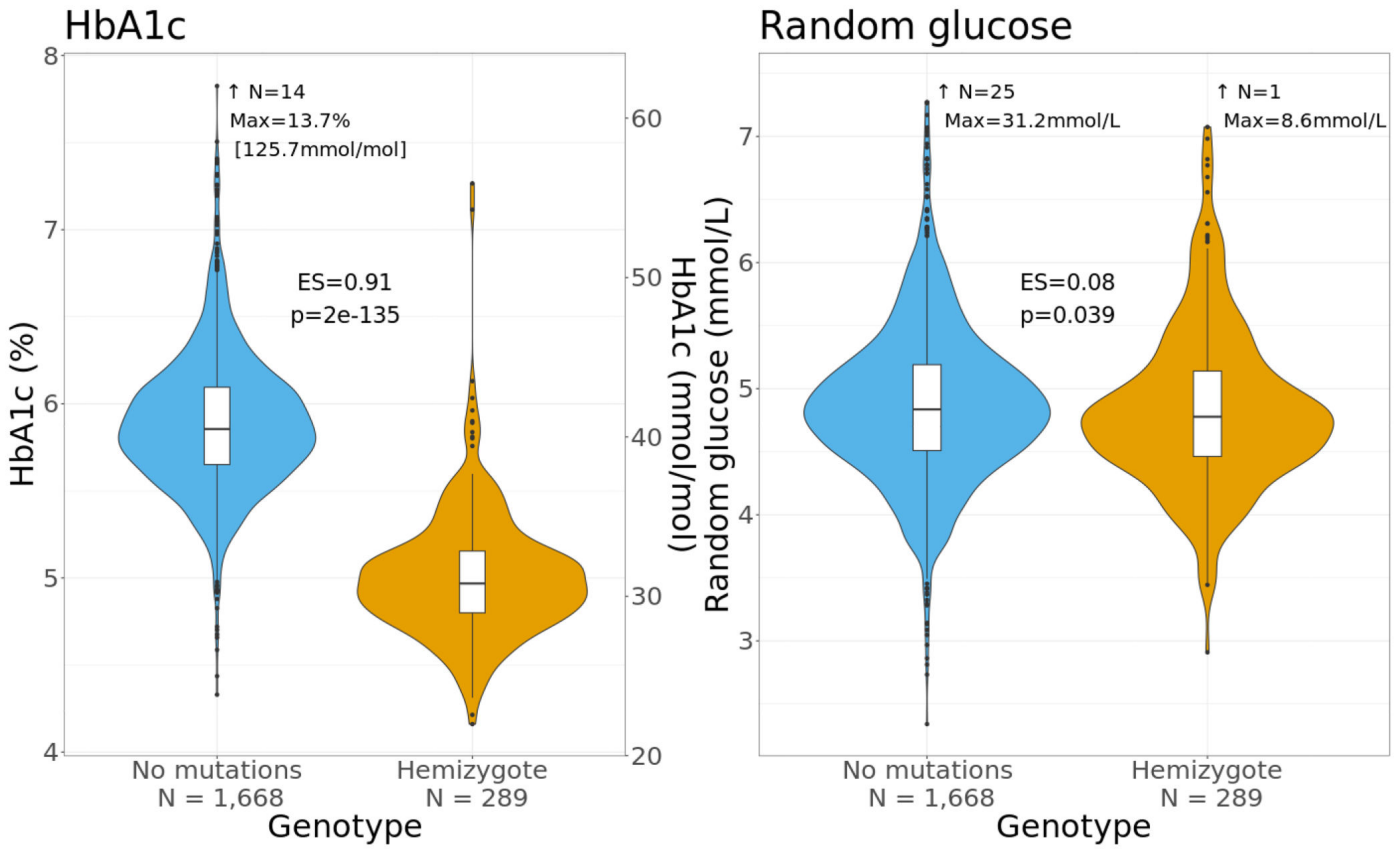

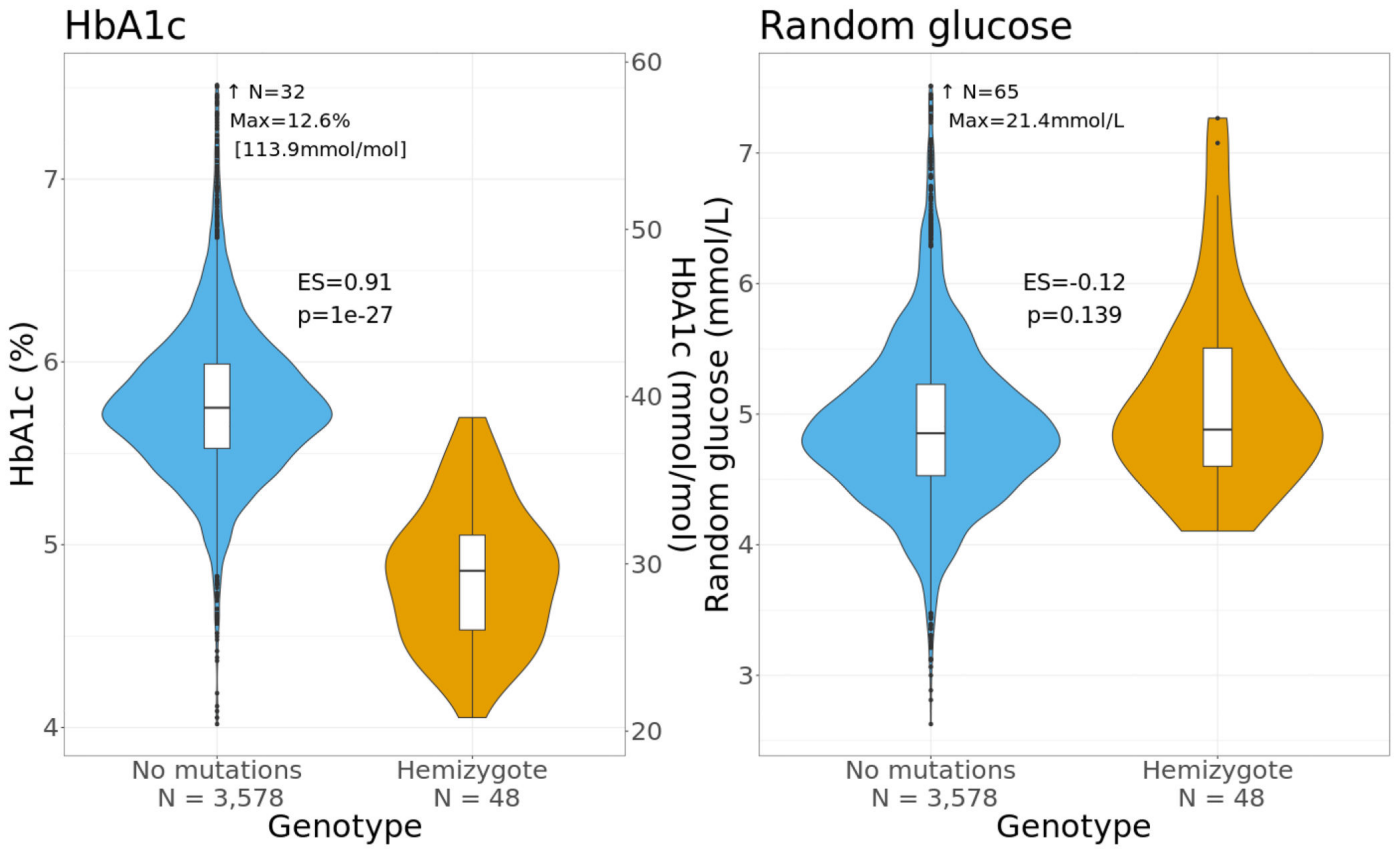

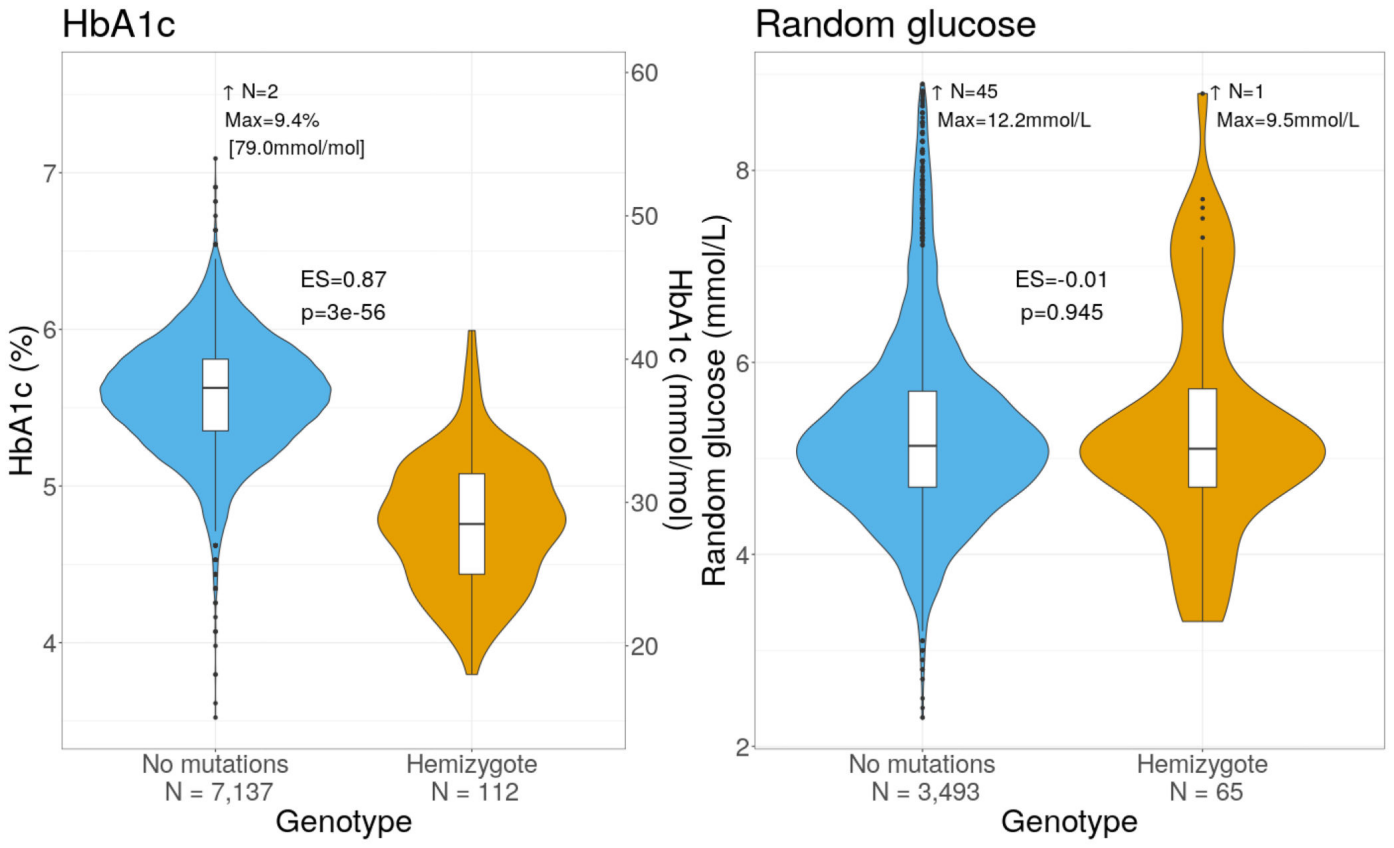
Violin and box plots for HbA1c and random glucose levels by carrier status for the G6PD deficiency (a) rs1050828-T allele in Black male UK Biobank participants; (b) rs5030868-A allele in Asian male UK Biobank participants; and (c) rs5030868-A allele in South Asian male Genes & Health participants; all without diabetes. Effect sizes represent Cliff’s Delta estimates, and p-values are taken from Mann-Whitney-U tests; box plots show the median and interquartile range, and the whiskers show minimum and maximum; plots are truncated at median + 5 × (median absolute difference) with characteristics of truncated values given. ES: effect size, N: sample size.

We found Black and Asian males with a G6PD deficiency allele had considerably lower average HbA1c (0.9% [International Federation of Clinical Chemistry (IFCC): 10.0mmol/mol]) compared to non-carriers, while differences in average glucose were negligible (Figure 1), consistent with recent reports(12; 13). Lower HbA1c levels were also observed in those with a T2D diagnosis, while differences in average glucose were also much smaller (Figure S2). We note that in our population cohort, random glucose values were considerably lower than comparable HbA1c concentrations (Table S1). This discrepancy is likely due to the influence of time since last meal, as the mean fasting time in the UK Biobank was 3.8 hours (SD 2.4), with 74.5% of the cohort having fasted over 3 hours, which may explain the low glucose values observed compared to completely random values(28). Additionally, in non-white ethnic groups, HbA1c can overestimate blood glucose in the general non-G6PD deficient population (Table S3)(29). Similar results were observed in homozygous women however, consistent with random X chromosome inactivation, heterozygous women have an effect intermediate between homozygous carriers and homozygous non-carriers (Figures S3-S4). As HbA1c is used to guide treatment this suggests that undiagnosed G6PD deficiency in individuals with T2D could contribute to increased risk of complications.

**Figure 2:**
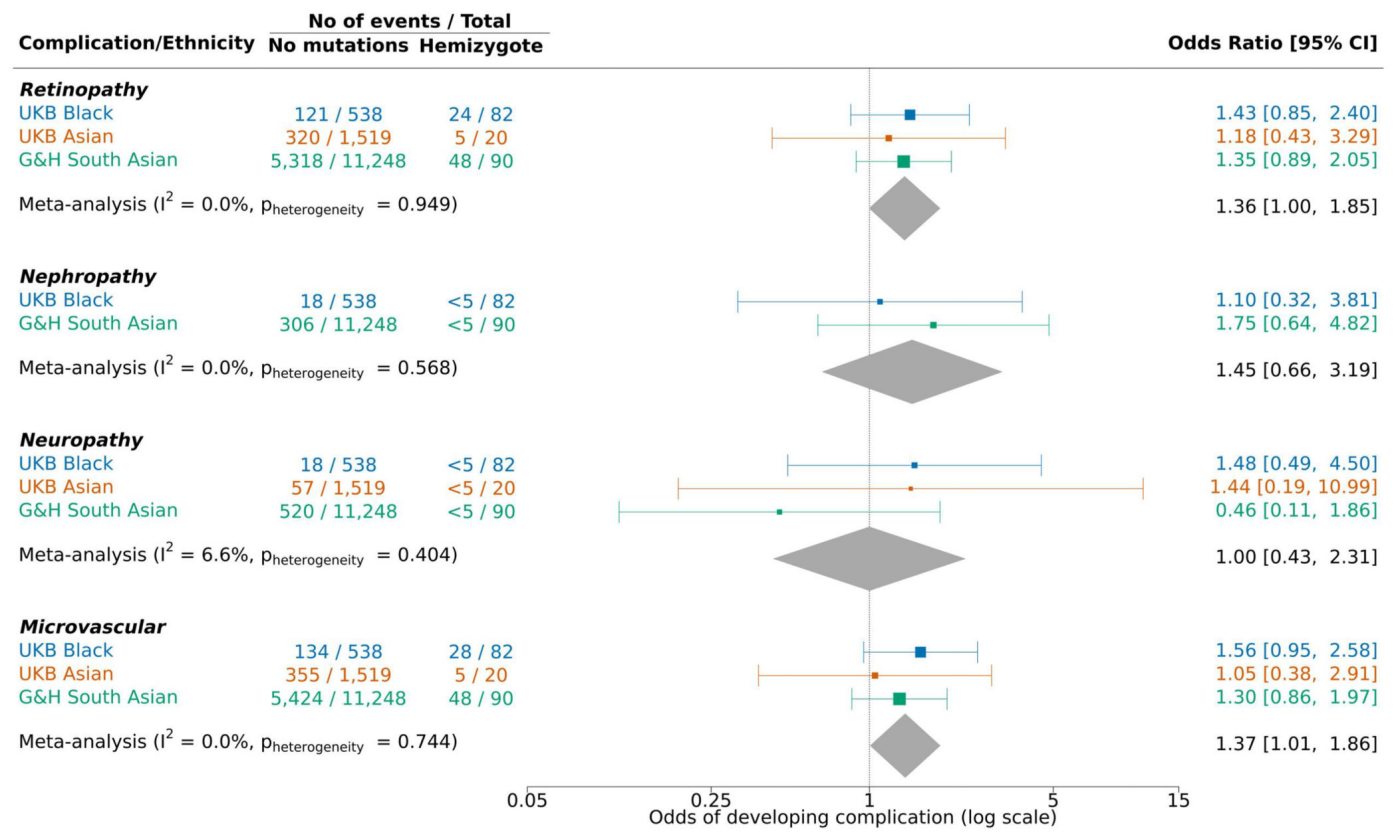

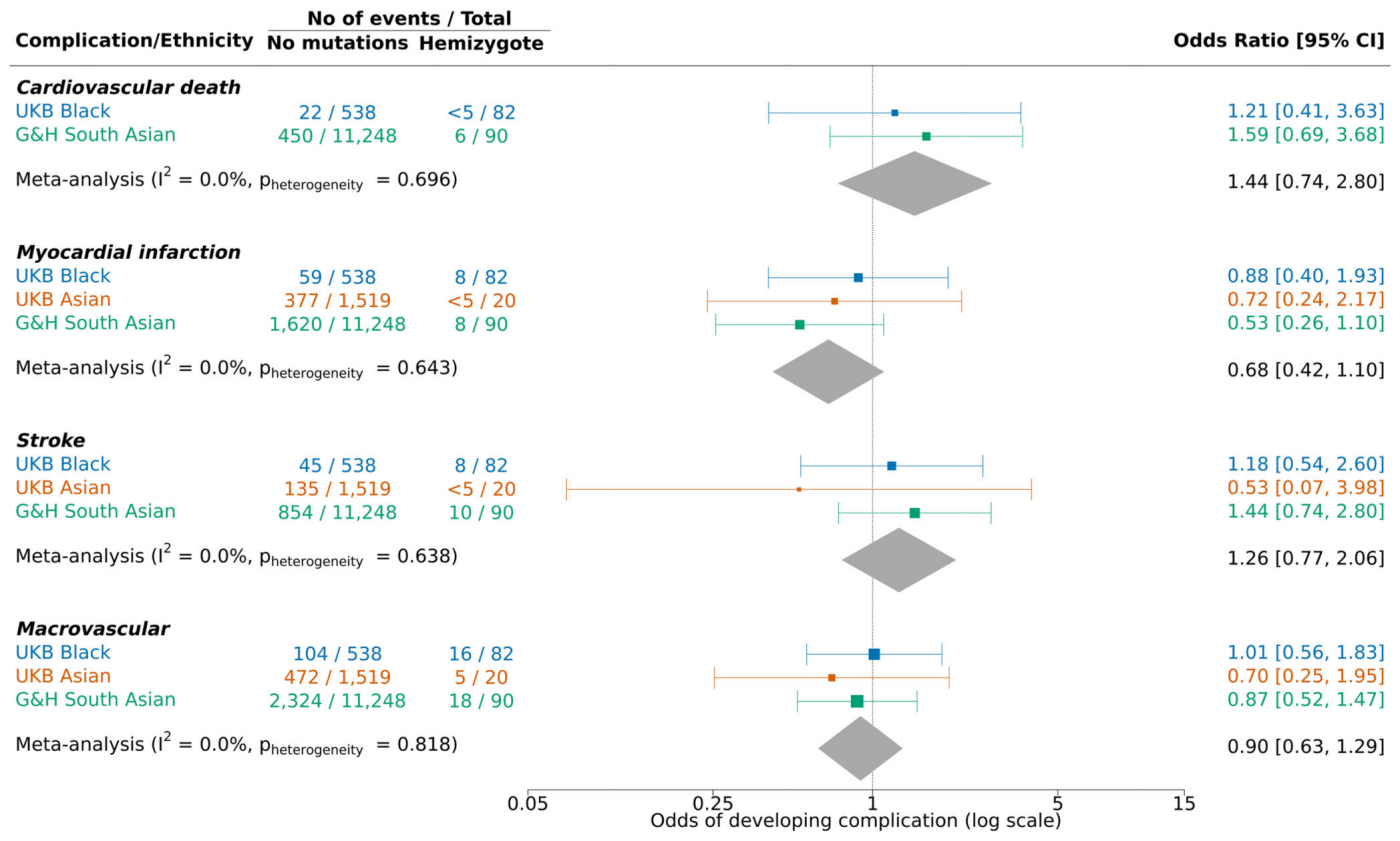

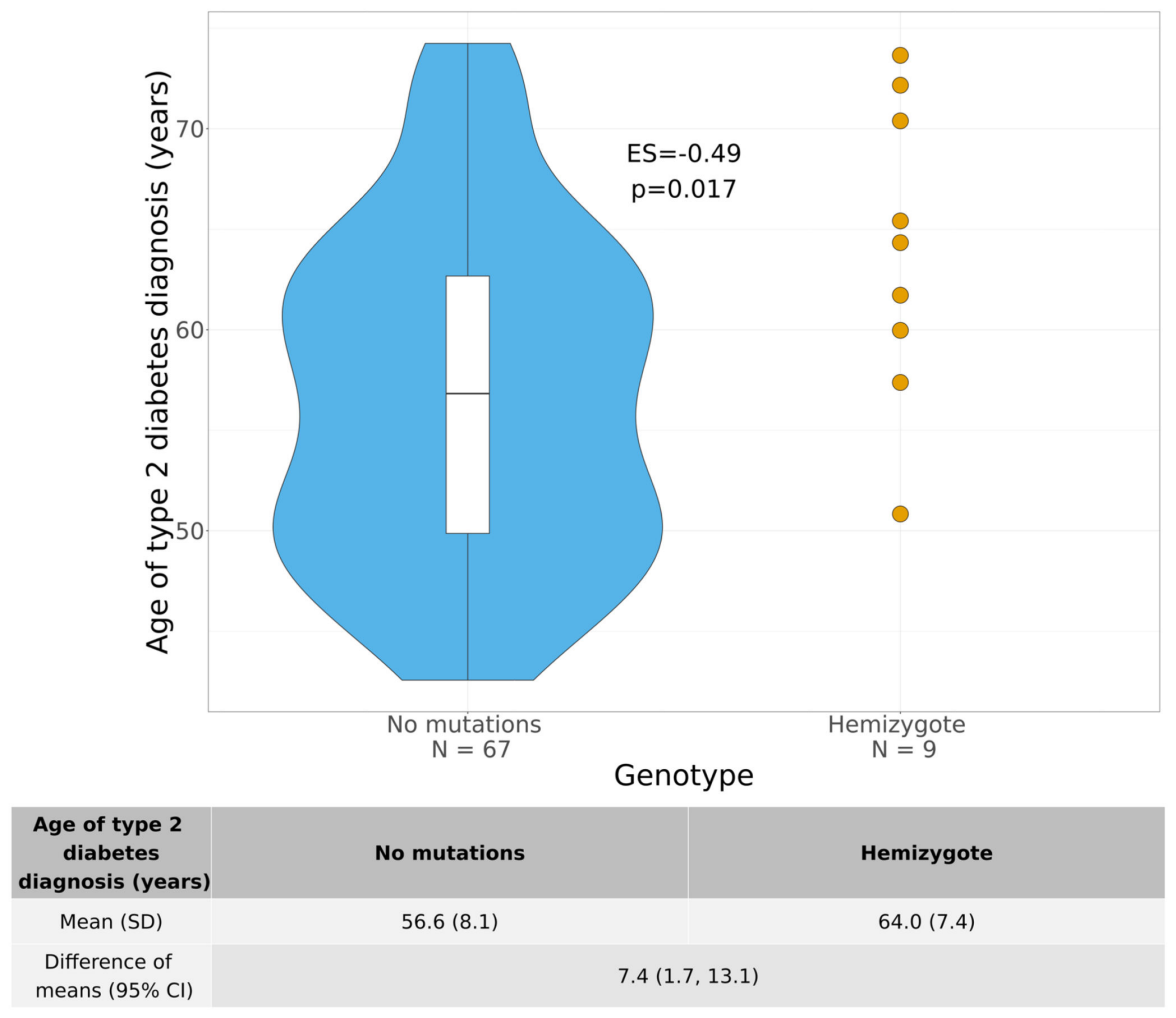

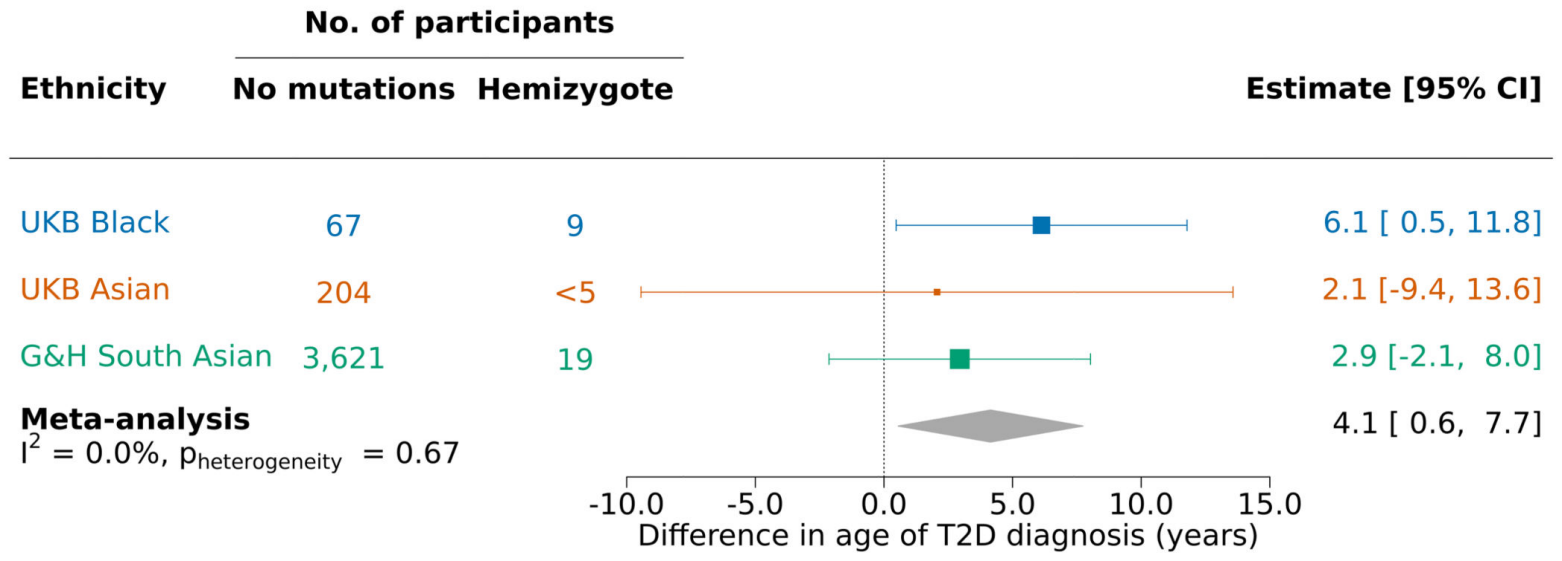
(a) Forest plot and random-effects meta-analysis of odds of developing microvascular complications in hemizygote vs non-carrier males diagnosed with type 2 diabetes at any time in the UK Biobank and Genes & Health ethnic groups. Nephropathy (N_NM_=39/1,519; N_H_=0/20) results for UK Biobank Asian group not included as model could not be fitted; sample sizes under 5 are hidden according to cohort guidelines. UKB: UK Biobank, G&H: Genes & Health, CI: confidence interval, N_NM_: no mutations sample size, N_H_: hemizygote sample size. (B) Forest plot and random-effects meta-analysis of odds of developing macrovascular complications in hemizygote vs non-carrier males diagnosed with type 2 diabetes at any time in the UK Biobank and Genes & Health ethnic groups. Cardiovascular death (N_NM_=110/1,519; N_H_=0/20) results for UK Biobank Asian group not included as model could not be fitted. (C) Violin and box plots for age of type 2 diabetes diagnosis by carrier status of the Asahi G6PD deficiency rs1050828-T allele in Black male UK Biobank participants. Effect size represents Cliff’s Delta estimate, and p-value is taken from Mann-Whitney-U test; the mean (SD) age at type 2 diabetes diagnosis and difference of means (95% CI) are shown in the table; box plots show the median and interquartile range, and the whiskers show minimum and maximum. ES: effect size, SD: standard deviation. (D) Forest plot and random-effects meta-analysis of adjusted difference in age of type 2 diabetes diagnosis between G6PD deficiency allele carriers versus non-carriers in males diagnosed after 2011 in the UK Biobank and Genes & Health ethnic groups. Adjusted difference in age estimates correspond to the coefficients from linear regression models adjusting for self-reported sub-ethnicity and electronic health record source of age. T2D: type 2 diabetes.

Given the lower levels of HbA1c in *G6PD* carriers with a T2D diagnosis which could lead to delays in treatment escalation, we sought to investigate the effect of G6PD deficiency allele carrier status on diabetes complications. The odds of developing microvascular complications were 1.37 [95% CI: 1.01, 1.86] times higher in male G6PD deficiency allele carriers than non-carriers (Figure 2a). Results were driven by effects on retinopathy, but lacked power for nephropathy and neuropathy (<5 carriers with these complications in each cohort ethnic group), the latter of which is known to be affected by ill-defined phenotyping in the UK Biobank(30). Results were inconclusive for macrovascular complications and women (Figures 2b, S5a-b), but overall had a consistent direction of effect with published results(12; 13).

Since HbA1c became the routine test to diagnose diabetes in the UK in 2011, we sought to assess the impact of undiagnosed G6PD deficiency on T2D age-at-diagnosis after 2011(23–25). Amongst Black males, carriers of G6PD deficiency alleles were on average 7.4 years [95% CI: 1.7, 13.1] older at diagnosis compared to non-carriers (Figure 2c), reflecting a delay in diagnosis, although caution should be exercised due to the small number of carriers (N=9). Sample sizes were modest but results were consistent in Asians (UK Biobank: <5 carriers, 204 non-carriers; Genes & Health: 19 carriers, 3,621 non-carriers), with meta-analysis across ethnicities and variants showing a 4.1-year [95% CI: 0.6,7.7] difference in age at diagnosis (Figure 2d). In female heterozygous carriers, effects are attenuated due to X-inactivation (Figure S5c).

Next, we compared the clinical characteristics before T2D diagnosis of G6PD deficiency allele carriers and non-carriers who went on to be diagnosed with T2D and found them to be clinically similar (Table S4). This suggests that despite similar clinical risk factors, low HbA1c levels may have been falsely reassuring and contributed to the delays in T2D diagnosis in men with G6PD deficiency.

As an example of how this could impact care pathways, we simulated two example Black African ethnicity male individuals of age 40 undergoing a T2D risk assessment, as is routinely offered in many countries, e.g. the NHS Health Check visit(27). Both individuals have matching high-risk factors: they are moderate smokers with body mass index = 31kg/m^2^, Townsend deprivation index = 3, with a family history of diabetes, treated for hypertension and with a history of statin use. These clinical features would give both individuals the same high 10-year risk of T2D of 48.6% based on QDiabetes-2018 score A. Based on this score, or the clinical risk factors alone, clinicians would likely obtain an HbA1c test for both. For one individual we assigned them an HbA1c measurement corresponding to the average HbA1c levels of male UK Biobank participants of Black ethnicity with no copies of the Asahi G6PD deficiency rs1050828-T allele (6.2% [IFCC: 44.6mmol/mol]) who did not have diabetes at baseline assessment but went on to develop diabetes. For the second individual, we assigned them an HbA1c of 5.6% [IFCC:37.9mmol/mol] corresponding to the average HbA1c of hemizygote carriers who did not have diabetes at baseline assessment but went on to develop diabetes. When the resulting HbA1c test result is incorporated into the subsequent QDiabetes-2018 score C, the individual carrying the *G6PD* variant would obtain a 41% lower risk estimate [30% vs 71%], based on the mean HbA1c from the *G6PD* carrier population. In contrast, the non-carrier with HbA1c of 6.2% [IFCC: 44.6mmol/mol] would be classified as having prediabetes in the UK based on recommendations by the International Expert Committee (HbA1c 6.0-6.4% [IFCC: 42-47mmol/mol])(31). Consequently, carriers and non-carriers would receive different care, with the non-carrier’s prediabetes diagnosis leading to an escalation in care involving T2D prevention interventions and yearly HbA1c screening(23).

**Figure 3:**
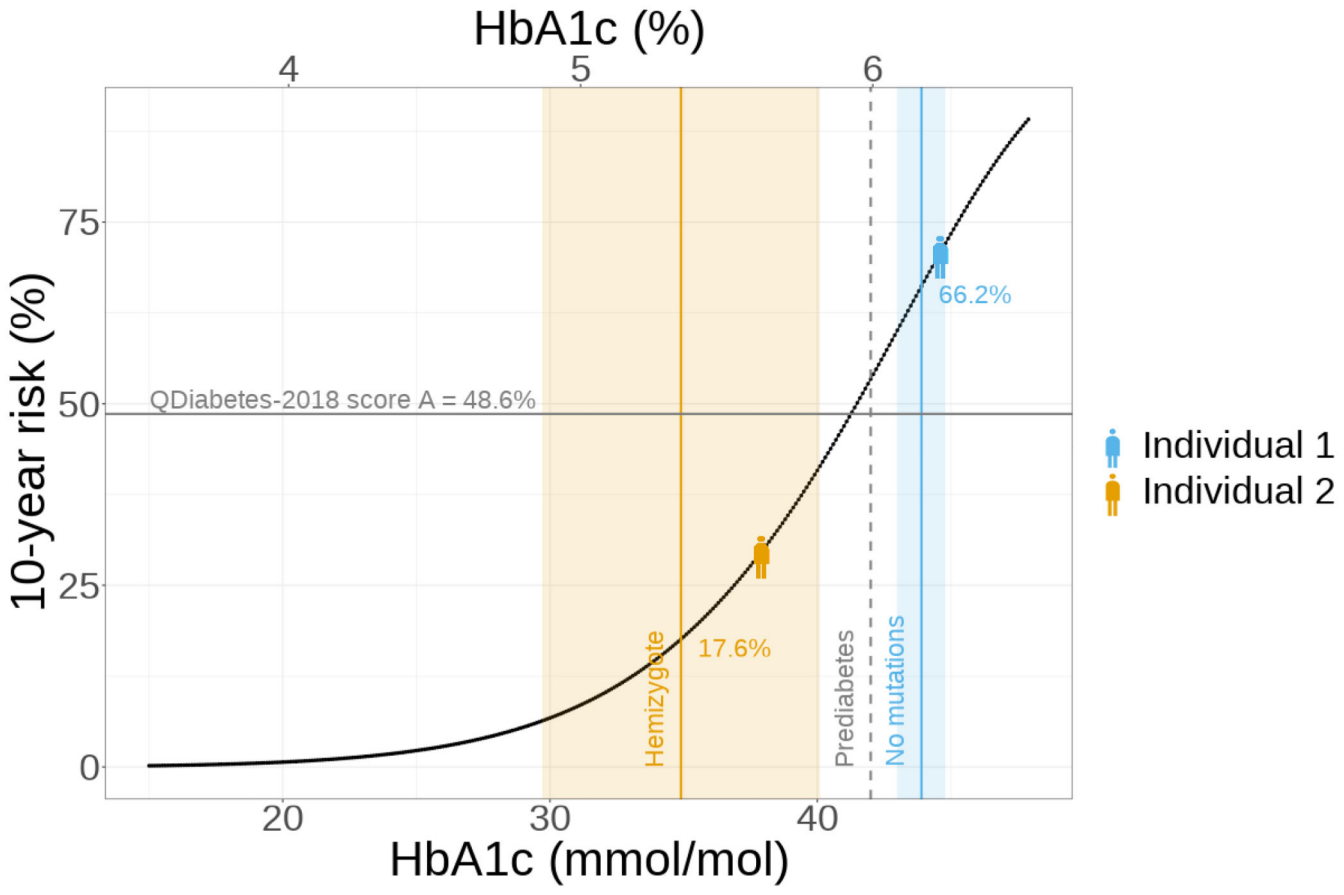

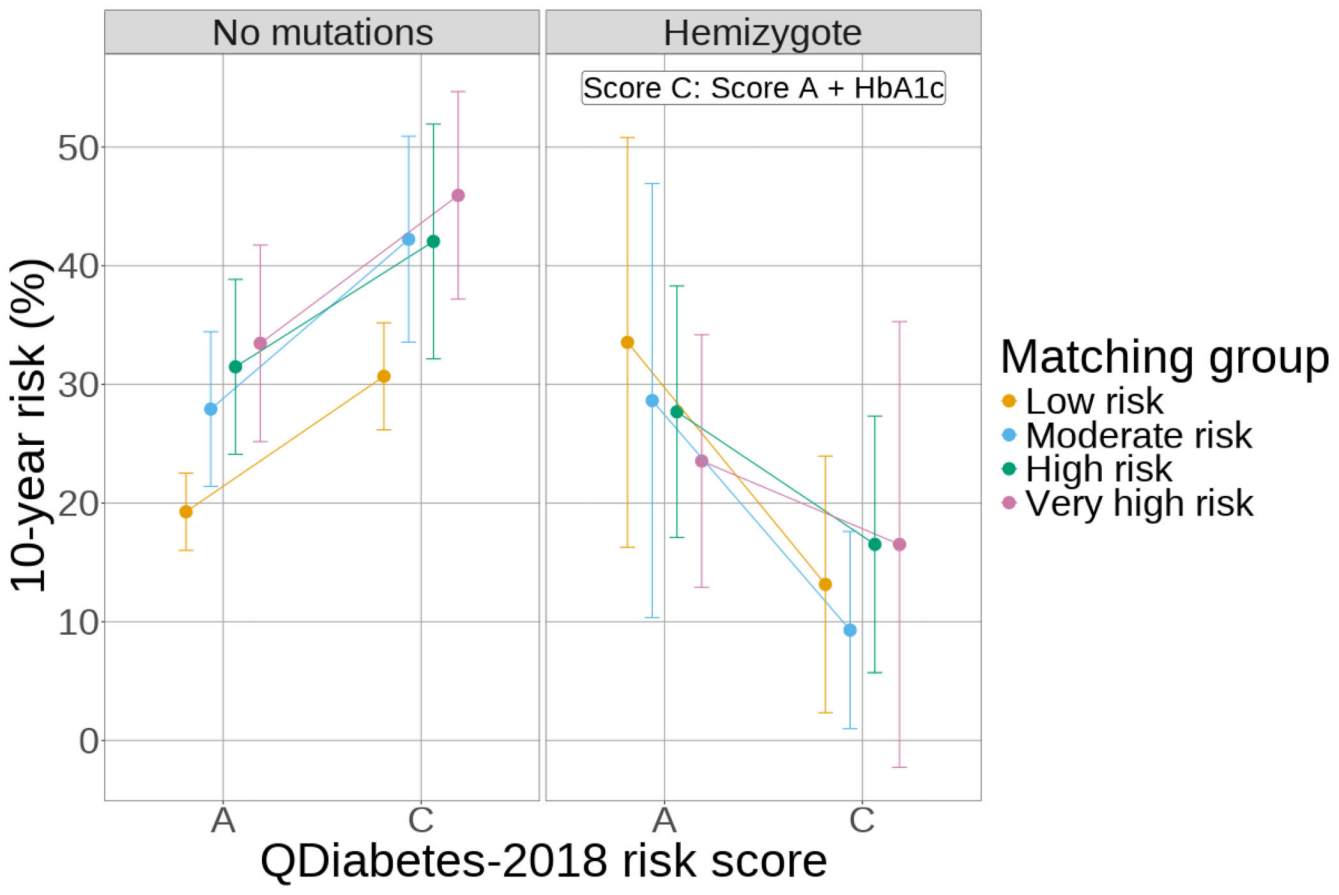
(a) 10-year risk of diabetes according to QDiabetes-2018 score C for example individual with varying HbA1c levels. Individuals 1 and 2 represent example 40-year-olds at NHS Health Check appointment who share the same risk variables for QDiabetes-2018 score A, but differ in their HbA1c levels to reflect the mean HbA1c for male UK Biobank participants of Black ethnicity with no copies of the G6PD deficiency rs1050828-T allele (Individual 1) and hemizygote carriers (Individual 2). Solid grey line represents the QDiabetes-2018 score A; orange line – mean HbA1c for male UK Biobank participants of Black ethnicity matched with Individual 1 (matching based on risk variables used in QDiabetes-2018 score A) and hemizygote carriers (N=6) for the G6PD deficiency rs1050828-T allele; blue line – same for participants matched with Individual 2 and with no copies of the G6PD deficiency rs1050828-T allele (N=24); shading – 95% confidence intervals; dashed line – diagnostic threshold for prediabetes. (b) 10-year risk of diabetes according to QDiabetes-2018 scores A and C for male UK Biobank participants of Black ethnicity with high-risk QDiabetes-2018 score A (>=5.6%) diagnosed with type 2 diabetes after baseline centre visit. Carrier and non-carrier participants have been matched into four groups based on their risk variables used in QDiabetes-2018 score A (from low to very high risk). Points represent the mean risk, and error bars the 95% confidence interval.

The clinical impact of carrying a *G6PD* variant is further demonstrated when observing its impact on QDiabetes-2018 C scores in our study population (Figure 3a). In UK Biobank men of Black ethnicity with and without G6PD deficiency, we observed that in all matched groups of risk factors, carriers had a lower risk score C compared to score A, while non-carriers obtained a higher risk score C (Figure 3b; Table S5). We performed similar analysis matching on QDiabetes-2018 score A instead of clinical risk factors and the results are similar (Figure S6). As risk score C includes information on HbA1c levels and is thought to be more accurate for diabetes risk prediction, these results imply male carriers are given a falsely reassuring low risk of future diabetes.

Compared to QDiabetes-2018 score A, the inclusion of HbA1c in score C worsened the 10-year prediction of T2D as assessed by NRI, with a 75.0% reduction in true-positive rate [95% CI: 61.3, 88.7] in UK Biobank male *G6PD* carriers of Black ethnicity with T2D (Figure S7). In comparison, the corresponding reduction in true-positive rate in non-carriers was 27.7% [95% CI: 22.4, 33.0]. The predictive performance of score C compared to score A in Black males, both with and without T2D, was lower in *G6PD* carriers (NRI 22.4% [95% CI: 8.7, 36.1]) compared to non-carriers (NRI 40.1% [95% CI: 34.4, 45.8]). These differences were generally consistent, albeit underpowered, in Asians (T2D in <5/45 carriers, 437/3,148 non-carriers) and females (Black: T2D in 43/475 heterozygotes, 162/1,301 non-carriers; Asian: 8/77 heterozygotes, 315/2,438 non-carriers).

## Discussion

Here, we have shown that G6PD deficiency is present in 1 in 7 Black and 1 in 63 Asian males in the UK, with only 1 in 50 men receiving a G6PD deficiency diagnosis. G6PD deficient men have a higher risk of diabetes-related microvascular complications (odds ratio 1.37 [95% CI: 1.01, 1.86]), and are diagnosed with T2D, on average, 4.1 years later than non-carriers. The increase in complications is likely to be a result of the late diagnosis and under-treatment of T2D guided by HbA1c, which is the WHO-recommended test to both diagnose and manage T2D in many healthcare systems worldwide. We have shown that the artificially low HbA1c levels in G6PD deficient men can falsely reassure clinicians and patients even in the presence of elevated risk factors for T2D, for example within the context of clinically used T2D risk prediction tools.

Strengths of our study include the use of longitudinal EHR data from two UK-based cohorts, including a cohort of South Asian ethnicity – a previously under-represented ethnic group in medical research – replicating our findings in both. We are the first study to demonstrate an age-of-diagnosis effect for T2D, as previous studies focused on delays in treatment and complications(12; 13), and to study the Mediterranean rs5030868 variant and find similar effects. This is also the first study to show how clinically recommended risk score calculators that include HbA1c test results can substantially underestimate the risk of developing T2D in *G6PD* carriers(21).

Limitations of the study include the use of UK-based cohorts only. Additionally, our sample sizes for ethnic minority groups, especially Black ethnicity, were low due to these groups being poorly represented in the UK Biobank. Primary care records are currently only available for half of the UK Biobank cohort, and so we chose not to use this data when defining binary outcome variables. When defining binary outcomes and risk factors in Genes & Health, EHR records alone had to be used, as there was no detailed questionnaire or interview at enrolment, and so we have assumed that a lack of record indicates a non-case, in line with other studies using EHR data(32).

Based on WHO guidance, the HbA1c test is used in 136 countries worldwide to diagnose diabetes(16), and is used for treat-to-target monitoring(33). The WHO currently estimates that G6PD deficiency affects more than 400 million individuals worldwide(4), and recommends routine screening in countries with a prevalence above 5%(7), nonetheless very few countries have implemented this recommendation(8). Consequently, the majority of G6PD deficient individuals are unaware of their condition. As a result, our findings are relevant to many countries with diverse populations, including the USA, as well as those with a high prevalence of G6PD deficiency in Caribbean, Latin America, Africa and Asia(34). This is of additional importance in Africa, where 25 countries use HbA1c as a diagnostic tool for diabetes(16), and where the implementation of this test has recently been suggested in other countries(35; 36). In addition to the two variants studied here, many other *G6PD* variants have been identified, including the *G6PD* Canton variant, which is prevalent in South East Asia(37). If these other *G6PD* variants have similar effects on HbA1c levels, then they could have a broader impact on these or other populations, and could further exacerbate this situation.

Even when guidelines, such as those set out by the American Diabetes Association(21), suggest the use of another test in addition to HbA1c, this may not mitigate against the problem of underdiagnosis. For example, if a HbA1c test is conducted first, then carriers could fall below the range required to suggest another test is needed and so may be missed. People from Black and ethnic minority backgrounds already face significant inequality in diabetes treatment in the UK and USA(38; 39). This study highlights the potential role of the HbA1c test in perpetuating and widening these inequalities, possibly leading to higher rates of complications and adverse outcomes for already marginalised groups.

In order to strengthen these results, future research should focus on larger cohorts of these ethnic minority groups in countries outside of the UK, where longitudinal data is available. For example, replication of our findings in additional biobanks (e.g. Million Veteran Program, All of Us) and other population cohorts with sequence and longitudinal data, especially from populations where G6PD deficiency has high prevalence (e.g. the Middle East, parts of Asia and Africa) would be highly beneficial to assess the validity of our findings in other world populations. The WHO recommends testing for G6PD deficiency in populations where prevalence is greater than 5%(7). In the UK, this could mean offering ethnicity-specific screening to all of those of Black African and Black Caribbean ethnicity, similar to screening for sickle cell disease(40). Alternatively, cost-effective strategies could be developed for implementation within the NHS to identify individuals for whom HbA1c is inaccurate. Future work should also investigate sickle cell disease, sickle cell trait and other haemoglobinopathies which frequently co-occur and may affect diagnosis of G6PD deficiency and impact on HbA1c.

## Conclusions

HbA1c is internationally recommended to diagnose and manage T2D. Undiagnosed G6PD deficiency is prevalent in minority ethnic groups and is associated with reassuring falsely low HbA1c levels. Awareness of this amongst clinicians and policymakers will be critical to address marked inequalities in T2D diagnosis and management, and to avoid further negative impact on T2D patients in other world populations where G6PD deficiency is common but not routinely screened.

## Supplementary Material

Supplementary material

## Figures and Tables

**Table 1:** Prevalence of the Asahi and Mediterranean G6PD deficiency variants and proportion of individuals with G6PD deficiency diagnosis in health records by self-reported ethnicity in male UK Biobank and Genes & Health participants. G6PD deficiency diagnosis results only presented for ethnicities where variants are most prevalent; sample sizes under 5 are hidden according to cohort guidelines. N: sample size.

G6PD deficiency variant	G6PD deficiency allele	Reference allele	Cohort	Ethnicity	Prevalence of G6PD deficiency allele (%)	No mutations			Hemizygote		
						N	G6PD deficiency diagnosis		N	G6PD deficiency diagnosis	
							N	%		N	%
**Asahi rs1050828**	T	C	UK Biobank	White	0.01	-			-		
				Black	15.14	2,651	<5	<0.19	473	<5	<1.06
				Asian	0.04	-			-		
				Mixed	1.46	-			-		
				Other	3.50	-			-		
			Genes & Health	South Asian	0.02	-			-		
**Mediterranean rs5030868**	A	G	UK Biobank	White	0.01	-			-		
				Black	0.06	-			-		
				Asian	1.56	5,351	0	0	85	<5	<5.88
				Mixed	0.39	-			-		
				Other	1.06	-			-		
			Genes & Health	South Asian	1.34	18,852	<5	<0.03	256	<5	<1.95

## Twitter summary

G6PD deficiency affects 1 in 7 Black and 1 in 63 Asian UK men. G6PD deficient men have a 4-year delay in type 2 diabetes diagnosis and 1.4 higher odds of microvascular complications than non-carriers.

## Article Highlights

- **Why did we undertake this study?**

G6PD deficiency is associated with lower HbA1c levels. However, the prevalence of this condition, and its effect on diabetes outcomes, had not been investigated in the UK.

- **What is the specific question(s) we wanted to answer?**

What is the prevalence of undiagnosed G6PD deficiency in the UK, and what is its impact on type 2 diabetes (T2D) diagnosis and progression to complications?

- **What did we find?**

One in 7 Black and 1 in 63 Asian UK males have G6PD deficiency, but <1 in 50 are diagnosed. G6PD deficient men are diagnosed with T2D 4.1-years later and have 1.37-higher odds of developing microvascular complications than non-carriers.

- **What are the implications of our findings?**

Highlighting the need to address inequalities in diabetes outcomes in *G6PD* carriers.
