# Supplementary material for: Undiagnosed G6PD deficiency in Black and Asian individuals is prevalent and contributes to health inequalities in type 2 diabetes diagnosis and complications
Source: Diabetes Care. Author manuscript; Available in PMC 2025 Nov 1. (PMC7618273; doi:10.2337/dc25-0556)
Supplement: Supplementary material [file EMS209618-supplement-Supplementary_material.pdf]

Undiagnosed G6PD deficiency in Black and Asian individuals is prevalent and contributes to health inequalities in type 2 diabetes diagnosis and complications

Authors

Susan Martin[1], Miriam Samuel[2], Daniel Stow[2], Alys M. Ridsdale[1], Ji Chen[1], Katherine G. Young[1], Harry D. Green[1], Genes & Health Research Team, Andrew T. Hattersley[1], Veline L'Esperance[2], Trevelyan J. McKinley[1], Sarah Finer[2] and Inês Barroso[1]*

1. Exeter Centre of Excellence for Diabetes Research (EXCEED), University of Exeter Medical School, Royal Devon & Exeter Hospital, Exeter, UK.
2. Wolfson Institute of Population Health, Queen Mary University of London, London, UK.

*Corresponding author: Inês Barroso, Exeter Centre of Excellence for Diabetes Research (EXCEED), University of Exeter Medical School, Royal Devon & Exeter Hospital, Exeter, UK (ines.barroso@exeter.ac.uk; 01392 408221)

Supplemental Material

For all statistical analyses, we excluded participants whose recorded sex was male but who had a heterozygous genotype for either of the X-chromosome *G6PD* variants of interest. Such individuals either had Klinefelter syndrome or represented an error in the sequencing or data recording. All statistical analyses were conducted using R software(1), and scripts are available for reproducibility at: https://github.com/susiemartin/G6PD_deficiency.

Whole exome sequencing

UK Biobank whole exome sequencing (WES) was performed by the Regeneron Genetics Center using the Illumina NovaSeq 6000 platform, with exomes captured using an altered version of the IDT xGen Exome Research Panel v.1.0, and is described in detail elsewhere(2). Genes & Health WES was performed on Oragene saliva DNA samples by the Broad Institute, who performed Standard Germline Exome V6 using Twist reagents and Illumina Novaseq sequencing, as well as basic quality control, and delivered crams with >85% bases at 20% target coverage(3). No other filtering was performed.

Self-reported ethnicity groupings

We grouped participants according to their self-reported ethnicity into five distinct groups based on the current UK census classifications from the Office for National Statistics(4), and excluded participants with missing self-reported ethnicity. Ethnicity groups were defined as follows:

- White – White, British, Irish, Any other white background.
- Black – African, Black or Black British, Caribbean, Any other Black background.
- Asian – Bangladeshi, Indian, Pakistani, Chinese, Any other Asian background.
- Mixed – Mixed, White and Asian, White and Black African, White and Black Caribbean, Any other mixed background.
- Other – Other ethnic group.

Clinical phenotype definitions

For the UK Biobank, electronic heath record (EHR) data was extracted from records on DNA Nexus(5). Further details on phenotype definitions and UK Biobank variables extracted using DNA Nexus are available for re-use and review at: https://github.com/susiemartin/G6PD_deficiency.

Diagnoses of glucose-6-phosphate dehydrogenase (G6PD) deficiency were extracted from primary and secondary care EHR data.

For the HbA1c and random glucose analysis, we used UK Biobank participants who had both measurements taken at the baseline assessment centre. A correction factor of $HbA1c_{corrected}$ = (0.9696*$HbA1c_{raw}$) + 3.3595 was applied to International Federation of Clinical Chemistry (IFCC) HbA1c readings (in mmol/mol) to account for differences in measurements taken at the assessment centre and in primary care, and any HbA1c measures greater than the maximum detectable value of 19.0% [IFCC: 184.0mmol/mol] were set to missing. For Genes & Health, we used participants who had a primary care record for either HbA1c or random glucose. The last valid recorded HbA1c or random glucose measurements were used for analysis, and these two measurements did not have to be taken on the same date to account for the primary care source of the Genes & Health data.

For UK Biobank, whole blood samples in ethylenediaminetetraacetic acid (EDTA) and silica clot accelerator (SST) collection tubes were stored at 4°C for up to 36 hours before processing and storage in liquid nitrogen(7; 8). Samples were stored for

4-10 years (median time for HbA1c and glucose measurements was 6.7 and 8.0 years respectively, in our cohort of 467,368 participants). HbA1c was then measured using HPLC analysis on a Bio-Rad VARIANT II Turbo, while glucose was measured using hexokinase analysis on a Beckman Coulter AU5800(8). The HbA1c and glucose assays were registered with UK National External Quality Assessment Services (NEQAS) External Quality Assurance (EQA) scheme and Wales External Quality Assessment Scheme (WEQAS) Mainline Chemistry EQA scheme respectively, both with International Organization for Standardization (ISO) 17025:2005 Quality Accreditation(9; 10). Algorithms were used to ensure that samples were tested in random order with respect to geography, date and time of sample collection(8). This allowed for day-to-day assay variation to be measured and corrected for, with the correction based on daily mean assay values.

For Genes & Health, all assays are performed in quality-assured laboratories under the UK Accreditation Service and adhering to ISO15189 standards(11). HbA1c testing in the UK follows IFCC standardisation and are reported in mmol/mol. Where historic values using Diabetes Control and Complications Trial (DCCT) standardisation (%) are used, these have been converted using the National Institutes of Health (NIH) formula: HbA1c[mmol/mol] = (HbA1c[%] - 2.15)*10.929(12). Where test results had incompatible units, i.e. units which could not be converted with a simple multiplication factor, these results were excluded as these were likely data entry errors. Manually curated minimum and maximum plausible value range for each trait were defined based on a priori clinical knowledge and reported ranges of these traits in UK Biobank (where available). It is important to note that these ranges were devised to exclude values which are likely due to

technical errors in sample processing or data entry, but to retain as much ‘real’ variation as possible. They are therefore somewhat deliberately broad. Trait values above or below the manually specified a priori limits of plausibility were excluded. These were as follows: Hba1c 1.4-19.0% [IFCC: 15.0-184.0mmol/mol], fasting glucose 0.6-45.0mmol/L, non-fasting glucose 0.6-45.0mmol/L. Test results obtained from before the age of 18, after the date of data extract, or with a missing date were excluded. The age at test was approximated from the month and year of birth stated by the volunteer in their baseline questionnaire. In some cases, a single test result can appear in the electronic health record multiple times due to duplicate data entry mechanisms. This was accounted for by excluding test results for the same volunteer with the exact identical value occurring within a 10-day rolling window.

When excluding individuals with conditions known to affect HbA1c readings, we specifically excluded individuals with a diagnosis of diabetes (any type), or who were pregnant or within six-weeks postpartum at time of HbA1c or random glucose measurement. For UK Biobank, this involved excluding those who met any of these criteria at time of baseline visit, and for Genes & Health, excluding those who met any of these criteria at the time the relevant reading was taken. However, we did not exclude those where their measurement was taken on the date of diabetes diagnosis, since treatment would not affect these readings. The above listed conditions were defined using self-reported, primary care (including prescription records) and secondary care records. Previously published self-reported and EHR code lists were used to define diabetes diagnosis and medications(13), and pregnancy outcomes(14).

For pregnancy status, pregnancy EHR codes were grouped into those corresponding to 12-week (ectopic or molar pregnancy), 24-week (miscarriage or termination) or 40-week gestation (delivery or stillbirth). Start date of pregnancy was estimated using:

$$\text{Start date} = \text{Episode end date} - W$$

where Episode end date is the date the outcome was recorded, and W = 12, 24 or 40 weeks depending on the pregnancy outcome recorded. Six-weeks postpartum was then added onto the episode end date to give the full interval where HbA1c values should be set to missing.

When comparing HbA1c and random glucose in individuals with a diagnosis of diabetes (any type), we only included individuals who met a strict definition of diabetes, and where the diagnosis was prevalent at time of measurement. This strict definition was similar to the definition used for exclusion described above, but did not include the use of prescription or self-reported medication records. As before, self-reported and EHR codes used to define diabetes included previously published code lists(13).

Age of type 2 diabetes (T2D) diagnosis was defined as the earliest age recorded in self-reported or primary care records since 1st January 2011. Secondary care records for age of diagnosis were not used as these would likely represent extreme presentations of symptoms to be diagnosed in a hospital setting, and so may reflect non-standard delayed diagnoses. When defining a diagnosis of T2D, we excluded individuals with any diagnosis of diabetes that was neither type 1 nor type 2 (e.g.

genetic or gestational diabetes), and also excluded those who were using insulin within one year of diagnosis. No limit on age of diagnosis was used to differentiate type 2 from type 1 diabetes here. Diagnoses and use of insulin was taken from self-reported, primary care (including prescription records) and secondary care records. EHR codes used to define T2D, non-type 1 or 2 diabetes, and insulin use included publicly available code lists(15).

For the QDiabetes-2018 risk scores, additional information on the following risk factors was required: body mass index (BMI), smoking status, townsend deprivation index (TDI), family history of diabetes in first-degree relative, history of hypertension, cardiovascular disease, gestational diabetes, polycystic ovary syndrome (PCOS), learning disabilities, schizophrenia or bipolar affective disorder, and history of use of corticosteroids, statins and second generation antipsychotics(16). When calculating the risk scores for both cohorts, BMI values below 20kg/m^2 were set to 20 and values above 40 were set to 40, in accordance with guidelines. In addition, truncation was applied to age (25 to 85 years) and TDI (-8 to 14) as required. Previously published self-reported and EHR code lists were used to define smoking status(17; 18); hypertension, blood pressure medications and PCOS(15; 18); cardiovascular disease(15; 18; 19); gestational diabetes; learning disabilities, schizophrenia and bipolar affective disorder(18); corticosteroids(20-22); statins(20; 23); and second generation antipsychotics(20; 24).

Risk factors were defined using self-reported baseline interview or HES records prior to baseline assessment centre visit for the UK Biobank. Primary care records were

not used for the UK Biobank as these are currently only available for half the cohort. Continuous measures and smoking status, categorised according to the number of reported cigarettes smoked daily, were taken from the baseline assessment centre visit. Family history of diabetes in a first-degree relative was defined using self-reported diabetes diagnosis in parent or sibling (fields 20107, 20110 and 20111). For Genes & Health, all risk factors were taken from EHR data, with age corresponding to the oldest age the BMI and HbA1c measurements were taken. For continuous measures, the last recorded measurement available was used, and the time period between these measures per individual ranged from 0 to 13.0 years. Smoking status and family history of diabetes were estimated using relevant EHR codes where recorded.

When comparing QDiabetes-2018 scores in participants with a diagnosis of T2D after risk factors were recorded, this was defined as after baseline assessment centre visit in UK Biobank, and after the oldest age the BMI and HbA1c measurements were taken in Genes & Health. All risk factors were defined using records taken on or before the date of type 2 diabetes diagnosis. T2D was defined as described above, but included only individuals whose earliest recorded date of diagnosis was after this defined date. In this case, dates of T2D diagnoses recorded in secondary care data were used. As before, EHR codes used to define diagnoses of T2D included publicly available code lists(15).

Diabetes-related complications were split into microvascular (retinopathy, nephropathy and neuropathy) and macrovascular (or major adverse cardiovascular

events (MACE)) (cardiovascular death, myocardial infarction and stroke). Each of these complications was defined using self-reported and secondary care records in the UK Biobank, as well as primary care records in Genes & Health. Primary care records were not used for the UK Biobank as these are currently only available for half the cohort. Presence of a complication was defined as also having a diagnosis of T2D at any time, where T2D was defined as described above using self-reported, primary and secondary care data. Publicly available EHR code lists were used to define the complications(18) and T2D(15).

For all clinical phenotypes, when defining binary outcomes and risk factors in Genes & Health, EHR records alone had to used, as there was no detailed questionnaire or interview at enrolment. As a result, we have assumed that lack of record indicates a non-case.

Comparison of HbA1c, glucose and age of T2D diagnosis across *G6PD* genotypes

HbA1c, random glucose and age of T2D diagnosis were compared across carrier status of G6PD deficiency alleles for each sex and ethnicity combination, using appropriate statistical tests and effect size estimates. We ran parametric checks on HbA1c, random glucose and age of T2D diagnosis independently for each sex- and ethnic-specific cohort. This involved checking for normality assumptions using density plots and quantile-quantile (Q-Q) plots, as well as Shapiro-Wilk tests for sample sizes <5,000 or Kolmogorov-Smirnov tests otherwise. The equality of

variance assumption was checked using F-tests in male cohorts, where only two genotypes are possible, and female cohorts containing more than one participant for only two of the three possible genotypes. For female cohorts containing more than one participant for each of the three possible genotypes, Bartlett's tests were used to check equality of variance. Plots and test results for these checks are given at: https://github.com/susiemartin/G6PD_deficiency.

For male cohorts and female cohorts containing participants for only two genotypes, if parametric checks were met, then two-sample t-tests were applied and Cohen's d effect size estimates calculated. If parametric checks failed, then Mann-Whitney-U tests were applied and Cliff's Delta effect size estimates calculated. For female cohorts containing participants for each of the three possible genotypes, if parametric checks were met, then one-way analysis of variance (ANOVA) was applied and Eta-squared effect size estimates calculated. If parametric checks failed, then Kruskal-Wallis tests were applied and Eta-squared effect size estimates calculated based on the Kruskal-Wallis test.

Regression models and meta-analysis

We compared age of T2D diagnosis, for those diagnosed since 2011, across carrier status of G6PD deficiency alleles in the UK Biobank using the following linear regression model:

$$\text{age of diagnosis}_i = \beta_0 + \beta_1 \text{genotype}_i + \beta_2 \text{source}_i + \beta_3 \text{ethnicity}_i + \varepsilon_i$$

where $i = 1, \ldots, \mathrm{N}$ are the participants, $\mathrm{age\ of\ diagnosis}$ is the minimum age of T2D diagnosis from GP and self-reported records, $\mathrm{genotype}$ is the number of G6PD deficiency alleles present for the genetic variant of interest (male carriers have been allocated a genotype of 1), source is the EHR source of age (GP or specific assessment centre), $\mathrm{ethnicity}$ is the self-reported sub-ethnicity, and ε_i is the residual error term ($\varepsilon_i \sim N(0, \sigma^2)$). The model was equivalent for the Genes & Health analysis, but did not include the source covariate as only GP records were used.

We compared presence of diabetes-related complications, for those diagnosed with T2D at any time, across carrier status of G6PD deficiency alleles in the UK Biobank using the following linear regression model:

$$\mathrm{complication}_i = \beta_0 + \beta_1 \mathrm{genotype}_i + \beta_2 \mathrm{ethnicity}_i + \varepsilon_i$$

where $\mathrm{complication}$ is the binary presence of a complication identified from HES or self-reported records, and i, $\mathrm{genotype}$, $\mathrm{ethnicity}$ and ε_i are defined as before. The model was equivalent for the Genes & Health analysis, but presence of a complication was only identified from HES records.

Diagnostic plots for these models for each cohort and ethnic group are available at: https://github.com/susiemartin/G6PD_deficiency. The definitions of both outcome variables are detailed in the 'Clinical phenotype definitions' section.

Random-effects meta-analysis, with restricted maximum likelihood (REML) heterogeneity variance estimate, was conducted across cohorts and ethnicity groups

for both regression model analyses. Where regression models could not be fitted due to small sample sizes, results were not presented or included in the corresponding meta-analyses.

QDiabetes-2018 score analysis and matching

QDiabetes-2018 risk scores were calculated using the QDiabetes package in R(25). Shared clinical risk factors of simulated example individuals were based on the average values for male UK Biobank participants of Black ethnicity. Only participants with non-missing information on all risk factors, including HbA1c, were included in the summary statistics of risk factors and calculation of QDiabetes-2018 scores A and C. The high-risk threshold for score A was 5.6%, and for score C was 22.9% in UK Biobank and 51.8% in Genes & Health, where the thresholds for score C were calculated using the cohort-specific approach described in the score guidelines(16).

Matching of individuals was conducted using the MatchIt package in R(26), utilising the subclassification approach with four subclasses selected. This ensured that matched subclasses were distinct from each other, while also ensuring that sample sizes were greater or equal to 5 for each subclass, allowing us to present summary statistics in accordance with cohort guidelines. Male UK Biobank participants of Black ethnicity, with high-risk score A (>=5.6%) diagnosed with T2D after baseline centre visit, were matched across carrier status for G6PD deficiency alleles, based on (a) score A and (b) risk variables used in score A. In each case, subclasses were ordered from low to very high risk. When matching participants to the two simulated example individuals, we included these two individuals in our cohort before

conducting matching as described above, based on risk variables used in score A. We then selected participants in the same matched group as each example individual. Diagnostic plots to assess balance after subclassification matching are available at: https://github.com/susiemartin/G6PD_deficiency.

Net reclassification index

We compared performance of QDiabetes-2018 scores A and C using categorical net reclassification index (NRI) in UK Biobank participants of Black and Asian ethnicity. NRI was calculated using the following equation(27):

$$NRI = NRI_{case} + NRI_{noncase}$$

with

$$NRI_{case} = P(\mathrm{up}|\mathrm{case}) - P(\mathrm{down}|\mathrm{case})$$

$$NRI_{noncase} = P(\mathrm{down}|\mathrm{noncase}) - P(\mathrm{up}|\mathrm{noncase})$$

where $P(\mathrm{up}|\mathrm{case})$ is the proportion of cases classed as low-risk using score A and high-risk using score C, $P(\mathrm{down}|\mathrm{case})$ is the proportion of cases classed as high-risk with score A and low-risk with score C, $P(\mathrm{down}|\mathrm{noncase})$ is the proportion of non-cases classed as high-risk with score A and low-risk with score C, and $P(\mathrm{up}|\mathrm{noncase})$ is the proportion of non-cases classed as low-risk with score A and high-risk with score C.

Individuals were classed as high-risk of developing T2D within 10 years if score A >=5.6%. The corresponding high-risk threshold for score C in this cohort was 22.9%,

which was calculated using the cohort-specific approach described in the score guidelines(16). T2D cases here were defined using the T2D definition described in the ‘Clinical phenotype definitions’ section. Here, ‘low risk’ refers to not being classed as high-risk using the above-described score thresholds.

Supplementary References

1. R Core Team. R: A Language and Environment for Statistical Computing. Vienna, Austria, R Foundation for Statistical Computing, 2020
2. Backman JD, Li AH, Marcketta A, Sun D, Mbatchou J, Kessler MD, Benner C, Liu D, Locke AE, Balasubramanian S, Yadav A, Banerjee N, Gillies CE, Damask A, Liu S, Bai X, Hawes A, Maxwell E, Gurski L, Watanabe K, Kosmicki JA, Rajagopal V, Mighty J, Jones M, Mitnaul L, Stahl E, Coppola G, Jorgenson E, Habegger L, Salerno WJ, Shuldiner AR, Lotta LA, Overton JD, Cantor MN, Reid JG, Yancopoulos G, Kang HM, Marchini J, Baras A, Abecasis GR, Ferreira MAR. Exome sequencing and analysis of 454,787 UK Biobank participants. Nature 2021;599:628-634
3. Narasimhan VM, Hunt KA, Mason D, Baker CL, Karczewski KJ, Barnes MR, Barnett AH, Bates C, Bellary S, Bockett NA, Giorda K, Griffiths CJ, Hemingway H, Jia Z, Kelly MA, Khawaja HA, Lek M, McCarthy S, McEachan R, O'Donnell-Luria A, Paigen K, Parisinos CA, Sheridan E, Southgate L, Tee L, Thomas M, Xue Y, Schnall-Levin M, Petkov PM, Tyler-Smith C, Maher ER, Trembath RC, MacArthur DG, Wright J, Durbin R, van Heel DA. Health and population effects of rare gene knockouts in adult humans with related parents. Science 2016;352:474-477
4. Ethnic group classifications: Census 2021 [article online], 2021. Available from https://www.ons.gov.uk/census/census2021dictionary/variablesbytopic/ethnicgroupnationalidentitylanguageandreligionvariablescensus2021/ethnicgroup/classifications. Accessed 18 September 2023
5. UKBB Health Care Records [article online], 2024. Available from https://github.com/hdg204/UKBB.
6. Young KG, McDonald TJ, Shields BM. Glycated haemoglobin measurements from UK Biobank are different to those in linked primary care records: implications for combining biochemistry data from research studies and routine clinical care. Int J Epidemiol 2022;51:1022-1024
7. Elliott P, Peakman TC. The UK Biobank sample handling and storage protocol for the collection, processing and archiving of human blood and urine. Int J Epidemiol 2008;37:234-244
8. Biomarker assay quality procedures: approaches used to minimise systematic and random errors (and the wider epidemiological implications) V1.2 [article online], 2019. Available from https://biobank.ndph.ox.ac.uk/ukb/ukb/docs/biomarker_issues.pdf. Accessed 8 May 2025
9. UK Biobank Biomarker Enhancement Project – Companion Document to Accompany HbA1c Biomarker Data V1.0. [article online], 2018. Available from https://biobank.ndph.ox.ac.uk/showcase/showcase/docs/serum_hb1ac.pdf. Accessed 2 June 2025
10. UK Biobank Biomarker Project - Companion Document to Accompany Serum Biomarker Data V1.0. [article online], 2019. Available from https://biobank.ndph.ox.ac.uk/showcase/showcase/docs/serum_biochemistry.pdf. Accessed 2 June 2025
11. Jacobs BM, Stow D, Hodgson S, Zöllner J, Samuel M, Kanoni S, Bidi S, Walter K, Langenberg C, Dobson R, Finer S, Morton C, Siddiqui MK, Martin HC, Pietzner M, Mathur R, van Heel DA. Genetic architecture of routinely acquired blood tests in a British South Asian cohort. Nat Commun 2024;15:8929
12. Li Z, Calhoun P, Ruedy KJ, Beck RW. Concordance of Central Laboratory Hemoglobin A1c Measurements from Capillary Kits Compared to Venous Draws in the Insulin-Only Bionic Pancreas Pivotal Trial. Diabetes Technol Ther 2023;25:513-515
13. Young KG, McGovern AP, Barroso I, Hattersley AT, Jones AG, Shields BM, Thomas NJ, Dennis JM. The impact of population-level HbA(1c) screening on reducing diabetes diagnostic delay in middle-aged adults: a UK Biobank analysis. Diabetologia 2023;66:300-309
14. Minassian C, Williams R, Meeraus WH, Smeeth L, Campbell OMR, Thomas SL. Methods to generate and validate a Pregnancy Register in the UK Clinical Practice Research Datalink primary care database. Pharmacoepidemiol Drug Saf 2019;28:923-933

15. UK Biobank codelists [article online], 2024. Available from https://github.com/drkgyoung/UK_Biobank_codelists.
16. Hippisley-Cox J, Coupland C. Development and validation of QDiabetes-2018 risk prediction algorithm to estimate future risk of type 2 diabetes: cohort study. Bmj 2017;359:j5019
17. Havard A, Jorm LR, Lujic S. Risk adjustment for smoking identified through tobacco use diagnoses in hospital data: a validation study. PLoS One 2014;9:e95029
18. MULTIPLY initiative [article online], 2023. Available from https://github.com/Fabiola-Eto/MULTIPLY-Initiative.
19. Forbes H, Langan S. Clinical codelist - CVD ICD-10 codes. London School of Hygiene & Tropical Medicine, London, United Kingdom, 2018
20. Ritchie SC, Taylor HJ, Liang Y, Manikpurage HD, Pennells L, Foguet C, Abraham G, Gibson JT, Jiang X, Liu Y, Xu Y, Kim LG, Mahajan A, McCarthy MI, Kaptoge S, Lambert SA, Wood A, Sim X, Collins FS, Denny JC, Danesh J, Butterworth AS, Di Angelantonio E, Inouye M. Integrated clinical risk prediction of type 2 diabetes with a multifactorial polygenic risk score. medRxiv 2024;
21. Coding classifications used [article online], 2023. Available from https://digital.nhs.uk/coronavirus/treatments/methodology/coding-classifications-used.
22. Quint JK. Therapy codelist - Oral Corticosteroid codes. London School of Hygiene & Tropical Medicine, London, United Kingdom, 2020
23. Low and medium intensity statins by all Sub-ICB Locations [article online], 2023. Available from https://openprescribing.net/measure/statinintensity/.
24. Second generation antipsychotics, excluding long acting injection [article online], 2024. Available from https://github.com/opensafely/codelist-development/issues/116.
25. QDiabetes: A package to calculate risk of type 2 diabetes [article online], 2021. Available from https://cran.r-project.org/package=QDiabetes.
26. Ho D, Imai K, King G, Stuart E. MatchIt: Nonparametric Preprocessing for Parametric Causal Inference. Journal of Statistical Software 2011;42:1-28
27. Pencina MJ, D'Agostino RB, Sr., D'Agostino RB, Jr., Vasan RS. Evaluating the added predictive ability of a new marker: from area under the ROC curve to reclassification and beyond. Stat Med 2008;27:157-172; discussion 207-112

Table S1. Summary characteristics for the UK Biobank and Genes & Health. Categorical variables given as N (%) and continuous variables given as mean (standard deviation). N: sample size.

Table S2. Prevalence of the Asahi and Mediterranean G6PD deficiency variants and proportion of individuals with G6PD deficiency diagnosis in health records by self-reported ethnicity in female UK Biobank and Genes & Health participants. G6PD deficiency diagnosis results only presented for ethnicities where variants are most prevalent; sample sizes under 5 are hidden according to cohort guidelines. N: sample size.

Table S3. Summary of glycaemic traits and fasting time by ethnicity for the UK Biobank. Variables given as mean (standard deviation). N: sample size, IFCC: International Federation of Clinical Chemistry.

Table S4. Summary of risk variables included in QDiabetes-2018 scores A and C in (a) male and (b) female UK Biobank participants of Black and Asian ethnicity and Genes & Health participants of South Asian ethnicity, all with type 2 diabetes diagnosis after baseline visit or date of measurements. Male Genes & Health participants were not included as zero hemizygote carriers with type 2 diabetes diagnosis after date of measurements were present. Continuous variables are given as mean (standard deviation) and categorical variables given as N (%); sample sizes under 5 are hidden according to cohort guidelines. N: sample size, BMI: body mass index.

Table S5. Summary characteristics of matched groups of male UK Biobank participants of Black ethnicity with high-risk QDiabetes-2018 score A (>=5.6%) diagnosed with type 2 diabetes after baseline centre visit. Individuals have been matched according to their (a) risk variables used in QDiabetes-2018 score A and (b) QDiabetes-2018 score A (from low to very high risk), and across carrier status for the Asahi G6PD deficiency rs1050828-T allele.

Figure S1. Study plan.

Figure S2. Violin and box plots for HbA1c and random glucose levels by carrier status for the G6PD deficiency (a) rs1050828-T allele in Black male UK Biobank participants; (b) rs5030868-A allele in Asian male UK Biobank participants; and (c) rs5030868-A allele in South Asian male Genes & Health participants; all with diabetes. Effect sizes represent Cliff's Delta estimates, and p-values are taken from Mann-Whitney-U tests; box plots show the median and interquartile range, and the whiskers show minimum and maximum; plots are truncated at median + 5 × (median absolute difference) with characteristics of truncated values given. ES: effect size, N: sample size.

Figure S3. Violin and box plots for HbA1c and random glucose levels by carrier status for the G6PD deficiency (a) rs1050828-T allele in Black female UK Biobank participants; (b) rs5030868-A allele in Asian female UK Biobank participants; and (c) rs5030868-A allele in South Asian female Genes & Health participants; all without diabetes or pregnancy. UK Biobank Asian homozygotes (N<5) are not presented according to cohort guidelines; effect sizes represent Eta-squared estimates, and p-values are taken from Kruskal-Wallis tests; box plots show the median and interquartile range, and the whiskers show minimum and maximum; plots are truncated at median + 5 × (median absolute difference) with characteristics of truncated values given. ES: effect size, N: sample size.

Figure S4. Violin and box plots for HbA1c and random glucose levels by carrier status for the G6PD deficiency (a) rs1050828-T allele in Black female UK Biobank participants; (b) rs5030868-A allele in Asian female UK Biobank participants; and (c) rs5030868-A allele in South Asian female Genes & Health participants; all with diabetes. Groups with non-zero sample sizes under 5 are not presented according to cohort guidelines; effect sizes represent Eta-squared estimates, and p-values are taken from Kruskal-Wallis tests; box plots show the median and interquartile range, and the whiskers show minimum and maximum; plots are truncated at median + 5 × (median absolute difference) with characteristics of truncated values given. ES: effect size, N: sample size.

Figure S5. (a) Forest plot and random-effects meta-analysis of odds of developing microvascular complications for each additional G6PD deficiency allele in females diagnosed with type 2 diabetes at any time in the UK Biobank and Genes & Health ethnic groups. Neuropathy (N_{NM}=25/1,008; N_{HE}=0/22; N_{HO}=0/<5) results for UK Biobank Asian group not included as model could not be fitted; sample sizes under 5 are hidden according to cohort guidelines. UKB: UK Biobank, G&H: Genes & Health, CI: confidence interval, N_{NM}: no mutations sample size, N_{HE}: heterozygote sample size, N_{HO}: homozygote sample size.

(b) Forest plot and random-effects meta-analysis of odds of developing macrovascular complications for each additional G6PD deficiency allele in females diagnosed with type 2 diabetes at any time in the UK Biobank and Genes & Health ethnic groups.

(c) Forest plot and random-effects meta-analysis of adjusted difference in age of type 2 diabetes diagnosis per additional G6PD deficiency allele in females diagnosed after 2011 in the UK Biobank and Genes & Health ethnic groups. Adjusted difference in age estimates correspond to the coefficients from linear regression models adjusting for self-reported sub-ethnicity and electronic health record source of age. T2D: type 2 diabetes.

Figure S6. (a) 10-year risk of diabetes according to QDiabetes-2018 score C for example individual with varying HbA1c levels. Individuals 1 and 2 represent the simulated individuals described in **Figure 3a**, and have corresponding risk variables for QDiabetes-2018 score A. Solid grey line represents the QDiabetes-2018 score A; orange line – mean HbA1c for male UK Biobank participants of Black ethnicity matched with Individual 1 (matching based on having a QDiabetes-2018 score A >= 40%) and hemizygote carriers (N=4) for the G6PD deficiency rs1050828-T allele; blue line – same for participants matched with Individual 2 and with no copies of the G6PD deficiency rs1050828-T allele (N=23); shading – 95% confidence intervals; dashed line – diagnostic threshold for prediabetes.

(b) 10-year risk of diabetes according to QDiabetes-2018 scores A and C for male UK Biobank participants of Black ethnicity with high-risk QDiabetes-2018 score A (>=5.6%) diagnosed with type 2 diabetes after baseline centre visit. Carrier and non-carrier participants have been matched into four groups based on their QDiabetes-2018 score A (from low to very high risk). Points represent the mean risk, and error bars the 95% confidence interval.

Figure S7. Net reclassification index (NRI) of high risk according to QDiabetes-2018 score C (>=22.9%) compared to QDiabetes-2018 score A (>=5.6%) in UK Biobank (a) males and (b) females. Cases are those with incident type 2 diabetes diagnoses after baseline visit; non-cases are those without type 2 diabetes diagnoses at any time. Points represent the NRI, and error bars the 95% confidence interval from bootstrapping (N=1,000). Homozygotes are not presented as sample sizes are too small.

Genes & Health Research Team authorship

This sheet lists all individuals included in the Genes & Health Research Team authorship. It is expected that each individual is entered into 'Consortium' authorship using specific journal instructions to allow effective Pubmed searching by Genes & Health Research Team and by individual names. Please also ensure "Genes **&** Health" not "Genes and Health"

Eamonn Maher	e.maher@aston.ac.uk	Aston University
Shabana Chaudhary	s.chaudhary@qmul.ac.uk	Blizard Institute, Queen Mary University of London
Joseph Gafton	j.gafton@qmul.ac.uk	Blizard Institute, Queen Mary University of London
Karen A Hunt	k.a.hunt@qmul.ac.uk	Blizard Institute, Queen Mary University of London
Shapna Hussain	shapna.hussain@qmul.ac.uk	Blizard Institute, Queen Mary University of London
Kamrul Islam	k.islam@qmul.ac.uk	Blizard Institute, Queen Mary University of London
Mohammed Bodrul Mazid	m.b.mazid@qmul.ac.uk	Blizard Institute, Queen Mary University of London
Elizabeth Owor	e.owor@qmul.ac.uk	Blizard Institute, Queen Mary University of London
Jessry Russell	jessry.russell@qmul.ac.uk	Blizard Institute, Queen Mary University of London
Nishat Safa	n.safa@qmul.ac.uk	Blizard Institute, Queen Mary University of London
John Solly	j.solly@qmul.ac.uk	Blizard Institute, Queen Mary University of London
Marie Spreckley	m.spreckley@qmul.ac.uk	Blizard Institute, Queen Mary University of London
David A Van Heel	d.vanheel@qmul.ac.uk	Blizard Institute, Queen Mary University of London
Jan Whalley	j.whalley@qmul.ac.uk	Blizard Institute, Queen Mary University of London
Ishevanhu Zengeya	i.zengeya@qmul.ac.uk	Blizard Institute, Queen Mary University of London
Emily Mantle	e.mantle@qmul.ac.uk	Blizard Institute, Queen Mary University of London
Shaheen Akhtar	shaheen.akhtar@bthft.nhs.uk	Bradford Teaching Hospitals NHS Foundation Trust
Samina Ashraf	samina.ashraf@bthft.nhs.uk	Bradford Teaching Hospitals NHS Foundation Trust
Dan Mason	dan.mason@bthft.nhs.uk	Bradford Teaching Hospitals NHS Foundation Trust
John Wright	john.wright@bthft.nhs.uk	Bradford Teaching Hospitals NHS Foundation Trust
Daniel MacArthur	d.macarthur@garvan.au.org	Garvan Institute
Michael Simpson	michael.simpson@kcl.ac.uk	King's College London
Richard C Trembath	richard.trembath@kcl.ac.uk	King's College London
Gerome Breen	gerome.breen@kcl.ac.uk	Kings College London
Raymond Chung	raymond.chung@kcl.ac.uk	Kings College London
Sang Hyuck Lee	sang_hyuck.lee@kcl.ac.uk	Kings College London
Omar Asgar	omar.asghar1@nhs.net	Manchester University Hospitals
Joanne Harvey	joanne.henry@nihr.ac.uk	Manchester University Hospitals

Karen Tricker	karen.tricker@mft.nhs.uk	Manchester University Hospitals
Caroline Winckley	caroline.winckley@nihr.ac.uk	Manchester University Hospitals
Hanifa Khatun	hanifa.khatun@mft.nhs.uk	Manchester University Hospitals
Amna Asif	amna.asif@mft.nhs.uk	Manchester University Hospitals
Claudia Langenberg	claudia.langenberg@qmul.ac.uk	Precision Healthcare University Research Institute, Queen Mary University of London
Grainne Colligan	grainnec@safh.org.uk	Social Action for Health (charity)
Ceri Durham	cerid@safh.org.uk	Social Action for Health (charity)
Bill Newman	william.newman@manchester.ac.uk	University of Manchester
Ahsan Khan	cllrahsan.khan@walthamforest.gov.uk	Waltham Forest Council
Hilary Martin	hilary.martin@qmul.ac.uk	Wellcome Sanger Institute
Teng Heng	th13@sanger.ac.uk	Wellcome Sanger Institute
Matt Hurles	meh@sanger.ac.uk	Wellcome Sanger Institute
Vivek Iyer	vvi@sanger.ac.uk	Wellcome Sanger Institute
Georgios Kalantzis	gk18@sanger.ac.uk	Wellcome Sanger Institute
Vladimir Ovchinnikov	vo3@sanger.ac.uk	Wellcome Sanger Institute
Iaroslav Popov	ip13@sanger.ac.uk	Wellcome Sanger Institute
Klaudia Walter	kw8@sanger.ac.uk	Wellcome Sanger Institute
Panos Deloukas	p.deloukas@qmul.ac.uk	William Harvey Research Institute, Queen Mary University of London
David Collier	d.j.collier@qmul.ac.uk	William Harvey Research Institute, Queen Mary Universityof London
Ana Angel	a.cristinaangelgarcia@qmul.ac.uk	Wolfson Institute of Population Health, Queen Mary University of London
Saeed Bidi	saeed.bidi@qmul.ac.uk	Wolfson Institute of Population Health, Queen Mary University of London
Fabiola Eto	f.eto@qmul.ac.uk	Wolfson Institute of Population Health, Queen Mary University of London
Sarah Finer	s.finer@qmul.ac.uk	Wolfson Institute of Population Health, Queen Mary University of London
Chris Griffiths	c.j.griffiths@qmul.ac.uk	Wolfson Institute of Population Health, Queen Mary University of London
Sam Hodgson	s.hodgson@qmul.ac.uk	Wolfson Institute of Population Health, Queen Mary University of London
Benjamin M Jacobs	b.jacobs@qmul.ac.uk	Wolfson Institute of Population Health, Queen Mary University of London
Rohini Mathur	r.mathur@qmul.ac.uk	Wolfson Institute of Population Health, Queen Mary University of London
Caroline Morton	c.morton@qmul.ac.uk	Wolfson Institute of Population Health, Queen Mary University of London
Asma Qureshi	asmaa.qureshi@qmul.ac.uk	Wolfson Institute of Population Health, Queen Mary University of London
Stuart Rison	s.rison@qmul.ac.uk	Wolfson Institute of Population Health, Queen Mary University of London
Annum Salman	a.salman@qmul.ac.uk	Wolfson Institute of Population Health, Queen Mary University of London
Miriam Samuel	m.samuel@qmul.ac.uk	Wolfson Institute of Population Health, Queen Mary University of London
Moneeza K Siddiqui	moneeza.siddiqui@qmul.ac.uk	Wolfson Institute of Population Health, Queen Mary University of London

Daniel Stow	d.stow@qmul.ac.uk	Wolfson Institute of Population Health, Queen Mary University of London
Sabina Yasmin	sabina.yasmin@qmul.ac.uk	Wolfson Institute of Population Health, Queen Mary University of London
Julia Zöllner	j.zollner@qmul.ac.uk	Wolfson Institute of Population Health, Queen Mary University of London
Sheik Dowlut	s.dowlut@qmul.ac.uk	Wolfson Institute of Population Health, Queen Mary University of London

Table S1. Summary characteristics for the UK Biobank and Genes & Health. Categorical variables given as N (%) and continuous variables given as mean (standard deviation). N: sample size, IFCC: International Federation of Clinical Chemistry. *Fasting time is not available in Genes & Health.

Characteristic	UK Biobank (N = 467,368)	Genes & Health (N = 43,011)
**Sex**		
Male	213,889 (45.8%)	19,108 (44.4%)
Female	253,479 (54.2%)	23,903 (55.6%)
**Ethnicity**		
White	442,705 (94.7%)	-
Black	7,268 (1.6%)	-
Asian	10,506 (2.2%)	-
Mixed	2,720 (0.6%)	-
Other	4,169 (0.9%)	-
Bangladeshi	-	25,519 (59.3%)
Pakistani	-	17,153 (39.9%)
**Age (years)**	56.5 (8.1)	42.3 (12.0)
**Body mass index (kg/m^2)**	27.4 (4.8)	27.4 (4.9)
**HbA1c (% [IFCC: mmol/mol])**	5.7 (0.6) [IFCC: 38.4 (6.5)]	5.8 (1.0) [IFCC: 40.0 (10.7)]
**Random glucose (mmol/L)**	5.1 (1.2)	5.8 (2.2)
**Fasting time (hours)**	3.8 (2.4)	*
**G6PD deficiency**	19 (0.004%)	5 (0.01%)
**Diabetes (any type)**	50,178 (10.7%)	9,791 (22.8%)
**Type 2 diabetes (diagnosed since 2011)**	6,455 (1.4%)	7,190 (16.7%)
**Type 2 diabetes (diagnosed any time) with diabetes-related complications**		
Retinopathy	5,886 (1.3%)	4,803 (11.2%)
Nephropathy	636 (0.1%)	273 (0.6%)
Neuropathy	2,108 (0.5%)	509 (1.2%)
Cardiovascular death	3,017 (0.6%)	341 (0.8%)
Myocardial infarction	7,126 (1.5%)	702 (1.6%)
Stroke	3,871 (0.8%)	1,043 (2.4%)

Table S2. Prevalence of the Asahi and Mediterranean G6PD deficiency variants and proportion of individuals with G6PD deficiency diagnosis in health records by self-reported ethnicity in female UK Biobank and Genes & Health participants. G6PD deficiency diagnosis results only presented for ethnicities where variants are most prevalent; sample sizes under 5 are hidden according to cohort guidelines. N: sample size.

G6PD deficiency variant	G6PD deficiency allele	Reference allele	Cohort	Ethnicity	Prevalence of G6PD deficiency allele (%)	No mutations			Heterozygote			Homozygote		
						N	G6PD deficiency diagnosis		N	G6PD deficiency diagnosis		N	G6PD deficiency diagnosis	
							N	%		N	%		N	%
Asahi rs1050828	T	C	UK Biobank	White	0.01		-			-			-	
				Black	15.02	2,998	0	0	1,047	<5	<0.48	99	<5	<5.05
				Asian	0.02		-			-			-	
				Mixed	3.31		-			-			-	
				Other	3.38		-			-			-	
			Genes & Health	South Asian	0.02		-			-			-	
Mediterranean rs5030868	A	G	UK Biobank	White	0.02		-			-			-	
				Black	0.01		-			-			-	
				Asian	1.45	4,927	0	0	139	0	0	<5	0	0
				Mixed	0.27		-			-			-	
				Other	0.74		-			-			-	
			Genes & Health	South Asian	1.33	23,295	<5	<0.02	578	0	0	30	0	0

Table S3. Summary of glycaemic traits and fasting time by ethnicity for the UK Biobank. Variables given as mean (standard deviation).
N: sample size, IFCC: International Federation of Clinical Chemistry.

**Ethnicity**	**White**	**Black**	**Asian**	**Mixed**	**Other**
	**(N = 442,705)**	**(N = 7,268)**	**(N = 10,506)**	**(N = 2,720)**	**(N = 4,169)**
**HbA1c (% [IFCC: mmol/mol])**	5.6 (0.5) [IFCC: 38.2 (6.2)]	6.0 (0.9) [IFCC: 41.6 (9.9)]	6.0 (0.9) [IFCC: 42.3 (9.8)]	5.7 (0.7) [IFCC: 38.9 (7.1)]	5.9 (0.8) [IFCC: 40.6 (9.6)]
**Random glucose (mmol/L)**	5.1 (1.2)	5.1 (1.6)	5.4 (1.8)	5.1 (1.2)	5.3 (1.8)
**Fasting time (hours)**	3.8 (2.4)	5.1 (3.5)	4.2 (2.5)	4.1 (2.9)	4.5 (3.1)

Table S4. (a) Summary of risk variables included in QDiabetes-2018 scores A and C in male UK Biobank participants of Black and Asian ethnicity with type 2 diabetes diagnosis after baseline visit. Genes & Health participants were not included here as zero hemizygote carriers with type 2 diabetes diagnosis after date of measurements were present. Continuous variables are given as mean (standard deviation) and categorical variables given as N (%); sample sizes under 5 are hidden according to cohort guidelines. N: sample size, BMI: body mass index, IFCC: International Federation of Clinical Chemistry.

**Cohort**	**UK Biobank**			
**Ethnicity group**	**Black**		**Asian**	
**Carrier status**	**No mutations**	**Hemizygote**	**No mutations**	**Hemizygote**
**N**	163	24	448	<5
**Age (years)**	53.3 (8.4)	54.8 (7.1)	53.9 (8.1)	57.7 (5.0)
**BMI (kg/m^2)**	30.0 (4.9)	31.2 (6.5)	28.1 (4.2)	32.0 (2.9)
**Sub-ethnicity**				
Black African	77 (47.2%)	10 (41.7%)	-	-
Black Caribbean	83 (50.9%)	14 (58.3%)	-	-
Bangladeshi	-	-	16 (3.6%)	-
Pakistani	-	-	98 (21.9%)	-
Indian	-	-	219 (48.9%)	-
Chinese	-	-	37 (8.3%)	-
Other Asian	-	-	78 (17.4%)	-
Other	<5 (<3.1%)	0 (0%)	-	-
**Smoking status**				
Non	90 (55.2%)	14 (58.3%)	257 (57.4%)	-
Ex	42 (25.8%)	5 (20.8%)	106 (23.7%)	-
Light	14 (8.6%)	<5 (<20.8%)	28 (6.3%)	-
Moderate	10 (6.1%)	<5 (<20.8%)	29 (6.5%)	-
Heavy	7 (4.3%)	<5 (<20.8%)	28 (6.3%)	-
**Townsend deprivation index**	3.2 (3.4)	2.8 (3.7)	1.1 (3.3)	1.4 (5.3)
**Family history of diabetes**	58 (35.6%)	9 (37.5%)	240 (53.6%)	-
**History of treated hypertension**	82 (50.3%)	14 (58.3%)	200 (44.6%)	-
**History of cardiovascular disease**	24 (14.7%)	<5 (<20.8%)	86 (19.2%)	-
**History of learning disabilities**	0 (0%)	0 (0%)	0 (0%)	-
**History of schizophrenia or bipolar affective disorder**	<5 (<3.1%)	<5 (<20.8%)	<5 (<1.1%)	-
**History of corticosteroid use**	0 (0%)	0 (0%)	6 (1.3%)	-
**History of statin use**	37 (22.7%)	7 (29.2%)	151 (33.7%)	-
**History of use of 2nd generation antipsychotics**	<5 (<3.1%)	<5 (<20.8%)	0 (0%)	-
**HbA1c (% [IFCC: mmol/mol])**	6.1 (0.3) [IFCC: 43.5 (3.0)]	5.3 (0.4) [IFCC: 34.5 (4.0)]	6.0 (0.3) [IFCC: 42.2 (3.6)]	5.4 (0.3) [IFCC: 35.0 (4.3)]
**QDiabetes-2018 score A (%)**	24.2 (18.7)	28.4 (17.5)	31.5 (20.1)	49.2 (16.0)
**High-risk according to QDiabetes-2018 score A**	148 (90.8%)	24 (100%)	437 (97.5%)	-
**QDiabetes-2018 score C (%)**	36.1 (23.2)	13.9 (15.2)	38.3 (23.4)	18.1 (9.0)
**High-risk according to QDiabetes-2018 score C**	107 (65.6%)	6 (25.0%)	310 (69.2%)	-

Table S4. (b) Summary of risk variables included in QDiabetes-2018 scores A and C in female UK Biobank participants of Black and Asian ethnicity and Genes & Health participants of South Asian ethnicity with type 2 diabetes diagnosis after baseline visit or date of measurements. Continuous variables are given as mean (standard deviation) and categorical variables given as N (%); sample sizes under 5 are hidden according to cohort guidelines. N: sample size, BMI: body mass index, IFCC: International Federation of Clinical Chemistry.

**Cohort**	**UK Biobank**						**Genes & Health**		
**Ethnicity group**	**Black**			**Asian**			**South Asian**		
**Carrier status**	**No mutations**	**Heterozygote**	**Homozygote**	**No mutations**	**Heterozygote**	**Homozygote**	**No mutations**	**Heterozygote**	**Homozygote**
**N**	178	45	0	346	8	<5	625	8	<5
**Age (years)**	54.3 (8.2)	54.4 (7.0)	-	54.7 (7.8)	58.4 (7.9)	-	46.3 (10.9)	49.2 (13.1)	-
**BMI (kg/m^2)**	33.6 (6.3)	34.5 (6.5)	-	29.3 (5.1)	29.4 (3.7)	-	30.9 (5.5)	34.0 (5.1)	-
**Sub-ethnicity**									
Black African	56 (31.5%)	12 (26.7%)	-	-	-	-	-	-	-
Black Caribbean	120 (67.4%)	31 (68.9%)	-	-	-	-	-	-	-
Bangladeshi	-	-	-	<5 (<1.4%)	0 (0%)	-	440 (70.4%)	<5 (<62.5%)	-
Pakistani	-	-	-	74 (21.4%)	<5 (<62.5%)	-	185 (29.6%)	5 (62.5%)	-
Indian	-	-	-	191 (55.2%)	5 (62.5%)	-	-	-	-
Chinese	-	-	-	35 (10.1%)	0 (0%)	-	-	-	-
Other Asian	-	-	-	42 (12.1%)	0 (0%)	-	0 (0%)	0 (0%)	-
Other	<5 (<2.8%)	<5 (<11.1%)	-	-	-	-	-	-	-
**Smoking status**									
Non	133 (74.7%)	35 (77.8%)	-	320 (92.5%)	6 (75.0%)	-	621 (99.4%)	8 (100.0%)	-
Ex	27 (15.2%)	10 (22.2%)	-	15 (4.3%)	<5 (<62.5%)	-	0 (0%)	0 (0%)	-
Light	6 (3.4%)	0 (0%)	-	6 (1.7%)	0 (0%)	-	0 (0%)	0 (0%)	-
Moderate	<5 (<2.8%)	0 (0%)	-	<5 (<1.4%)	0 (0%)	-	<5 (<0.8%)	0 (0%)	-
Heavy	8 (4.5%)	0 (0%)	-	<5 (<1.4%)	<5 (<62.5%)	-	0 (0%)	0 (0%)	-
**Townsend deprivation index**	2.9 (3.2)	2.5 (3.9)	-	0.3 (3.1)	-0.6 (3.3)	-	7.0 (2.6)	6.8 (1.8)	-
**Family history of diabetes**	85 (47.8%)	27 (60%)	-	204 (59.0%)	<5 (<62.5%)	-	434 (69.4%)	<5 (<62.5%)	-
**History of treated hypertension**	103 (57.9%)	28 (62.2%)	-	146 (42.2%)	<5 (<62.5%)	-	257 (41.1%)	<5 (<62.5%)	-
**History of cardiovascular disease**	23 (12.9%)	7 (15.6%)	-	34 (9.8%)	<5 (<62.5%)	-	58 (9.3%)	<5 (<62.5%)	-
**History of gestational diabetes**	0 (0%)	0 (0%)	-	0 (0%)	0 (0%)	-	148 (23.7%)	<5 (<62.5%)	-
**History of polycystic ovary syndrome**	<5 (<2.8%)	0 (0%)	-	<5 (<1.4%)	0 (0%)	-	51 (8.2%)	<5 (<62.5%)	-
**History of learning disabilities**	0 (0%)	0 (0%)	-	0 (0%)	0 (0%)	-	<5 (<0.8%)	0 (0%)	-
**History of schizophrenia or bipolar affective disorder**	<5 (<2.8%)	<5 (<11.1%)	-	<5 (<1.4%)	0 (0%)	-	24 (3.8%)	0 (0%)	-
**History of corticosteroid use**	<5 (<2.8%)	<5 (<11.1%)	-	<5 (<1.4%)	<5 (<62.5%)	-	<5 (<0.8%)	0 (0%)	-
**History of statin use**	31 (17.4%)	12 (26.7%)	-	83 (24.0%)	<5 (<62.5%)	-	221 (35.4%)	<5 (<62.5%)	-
**History of use of 2nd generation antipsychotics**	<5 (<2.8%)	0 (0%)	-	0 (0%)	0 (0%)	-	32 (5.1%)	0 (0%)	-
**HbA1c (% [IFCC: mmol/mol])**	6.1 (0.3) [IFCC: 43.3 (3.2)]	5.9 (0.4) [IFCC: 40.8 (5.1)]	-	6.1 (0.2) [IFCC: 42.8 (3.1)]	5.8 (0.6) [IFCC: 40.0 (6.0)]	-	6.0 (0.3) [IFCC: 41.7 (3.8)]	5.8 (0.4) [IFCC: 40.3 (4.2)]	-
**QDiabetes-2018 score A (%)**	21.0 (13.5)	24.9 (16.7)	-	24.3 (17.1)	29.9 (16.3)	-	50.5 (28.3)	49.7 (32.1)	-
**High-risk according to QDiabetes-2018 score A**	162 (91.0%)	43 (95.6%)	-	315 (91.0%)	8 (100%)	-	607 (97.1%)	8 (100.0%)	-
**QDiabetes-2018 score C (%)**	34.7 (22.0)	29.5 (22.1)	-	33.7 (22.3)	27.5 (24.1)	-	53.2 (29.4)	40.6 (32.0)	-
**High-risk according to QDiabetes-2018 score C**	111 (62.4%)	24 (53.3%)	-	217 (62.7%)	5 (62.5%)	-	306 (49.0%)	<5 (<62.5%)	-

Table S5. Summary characteristics of matched groups of male UK Biobank participants of Black ethnicity with high-risk QDiabetes-2018 score A (>=5.6%) diagnosed with type 2 diabetes after baseline centre visit. Individuals have been matched according to their (a) risk variables used in QDiabetes-2018 score A and (b) QDiabetes-2018 score A (from low to very high risk), and across carrier status for the Asahi G6PD deficiency rs1050828-T allele. N: sample size.

**Group**	**Low risk**		**Medium risk**		**High risk**		**Very high risk**	
**Genotype**	**No mutations**	**Hemizygote**	**No mutations**	**Hemizygote**	**No mutations**	**Hemizygote**	**No mutations**	**Hemizygote**
**Matching - Risk variables used in QDiabetes-2018 score A**								
**N**	59	6	35	6	27	6	27	6
**Age (years)**	49.9 (7.0)	57.5 (6.5)	53.6 (8.2)	50.5 (5.9)	56.3 (7.2)	54.2 (7.7)	59.7 (8.3)	57.2 (7.6)
**BMI (kg/m^2)**	29.4 (4.3)	31.0 (3.2)	30.8 (3.7)	31.5 (5.4)	31.2 (4.6)	31.0 (3.0)	30.9 (5.0)	28.6 (3.6)
**Sub-ethnicity**								
Black African	38 (64.4%)	5 (83.3%)	18 (51.4%)	<5 (<83.3%)	9 (33.3%)	<5 (<83.3%)	6 (22.2%)	<5 (<83.3%)
Black Caribbean	19 (32.2%)	<5 (<83.3%)	17 (48.6%)	<5 (<83.3%)	18 (66.7%)	5 (83.3%)	21 (77.8%)	<5 (<83.3%)
Other	<5 (<8.5%)	0 (0%)	0 (0%)	0 (0%)	0 (0%)	0 (0%)	0 (0%)	0 (0%)
**Smoking status**								
Non	23 (39.0%)	<5 (<83.3%)	25 (71.4%)	<5 (<83.3%)	17 (63.0%)	5 (83.3%)	16 (59.3%)	<5 (<83.3%)
Ex	30 (50.8%)	<5 (<83.3%)	5 (14.3%)	0 (0%)	<5 (<18.5%)	<5 (<83.3%)	<5 (<18.5%)	0 (0%)
Light	<5 (<8.5%)	0 (0%)	<5 (<14.3%)	<5 (<83.3%)	5 (18.5%)	0 (0%)	<5 (<18.5%)	<5 (<83.3%)
Moderate	<5 (<8.5%)	0 (0%)	<5 (<14.3%)	0 (0%)	0 (0%)	0 (0%)	6 (22.2%)	<5 (<83.3%)
Heavy	<5 (<8.5%)	0 (0%)	<5 (<14.3%)	<5 (<83.3%)	<5 (<18.5%)	0 (0%)	<5 (<18.5%)	0 (0%)
**Townsend deprivation index**	4.3 (2.8)	5.8 (2.2)	4.1 (3.1)	3.1 (3.9)	2.2 (4.0)	2.4 (2.6)	1.3 (2.9)	0.0 (3.9)
**Family history of diabetes**	22 (37.3%)	<5 (<83.3%)	14 (40.0%)	<5 (<83.3%)	8 (29.6%)	<5 (<83.3%)	12 (44.4%)	<5 (<83.3%)
**History of treated hypertension**	17 (28.8%)	<5 (<83.3%)	21 (60.0%)	<5 (<83.3%)	18 (66.7%)	5 (83.3%)	23 (85.2%)	<5 (<83.3%)
**History of cardiovascular disease**	7 (11.9%)	0 (0%)	6 (17.1%)	<5 (<83.3%)	5 (18.5%)	<5 (<83.3%)	6 (22.2%)	0 (0%)
**History of learning disabilities**	0 (0%)	0 (0%)	0 (0%)	0 (0%)	0 (0%)	0 (0%)	0 (0%)	0 (0%)
**History of schizophrenia or bipolar affective disorder**	<5 (<8.5%)	0 (0%)	0 (0%)	0 (0%)	0 (0%)	0 (0%)	<5 (<18.5%)	<5 (<83.3%)
**History of corticosteroid use**	0 (0%)	0 (0%)	0 (0%)	0 (0%)	0 (0%)	0 (0%)	0 (0%)	0 (0%)
**History of statin use**	6 (10.2%)	<5 (<83.3%)	9 (25.7%)	0 (0%)	13 (48.1%)	<5 (<83.3%)	9 (33.3%)	<5 (<83.3%)
**History of use of 2nd generation antipsychotics**	<5 (<8.5%)	0 (0%)	0 (0%)	0 (0%)	0 (0%)	0 (0%)	<5 (<18.5%)	<5 (<83.3%)
**Matching - QDiabetes-2018 score A**								
**N**	55	6	27	6	33	6	33	6
**QDiabetes-2018 score A**	10.7 (3.0)	11.4 (3.3)	20.1 (2.4)	19.9 (2.2)	28.9 (3.3)	30.1 (3.4)	54.1 (16.8)	52.1 (16.3)

Figure S1. Study plan.

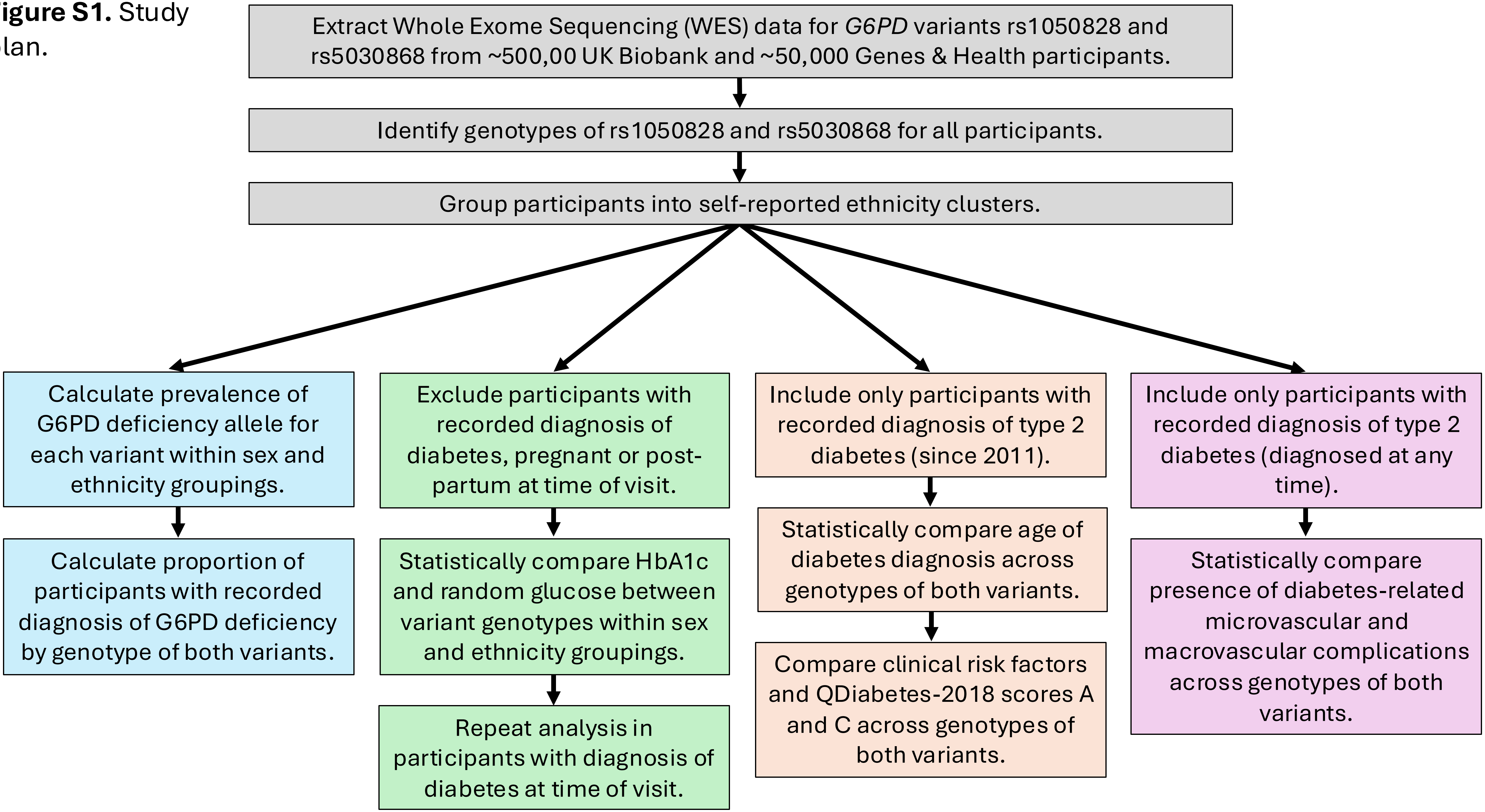

Figure S2a.

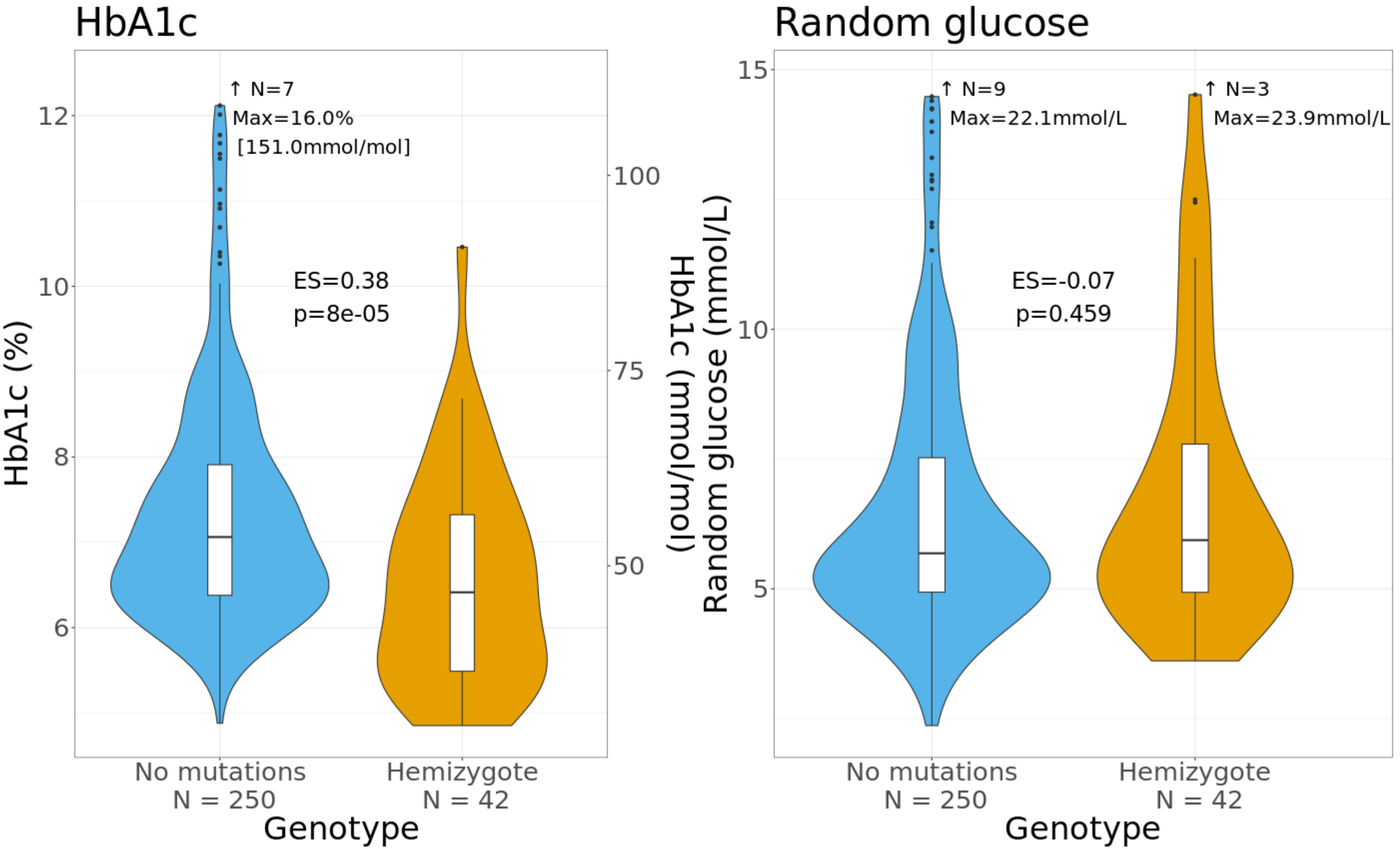
HbA1c
↑ N=7
Max=16.0%
[151.0mmol/mol]
ES=0.38
p=8e-05
HbA1c (%)
HbA1c (mmol/mol)
12
10
8
6
100
75
50
No mutations
N = 250
Hemizygote
N = 42
Genotype
Random glucose
↑ N=9
Max=22.1mmol/L
↑ N=3
Max=23.9mmol/L
ES=-0.07
p=0.459
Random glucose (mmol/L)
15
10
5
No mutations
N = 250
Hemizygote
N = 42
Genotype

Figure S2b.

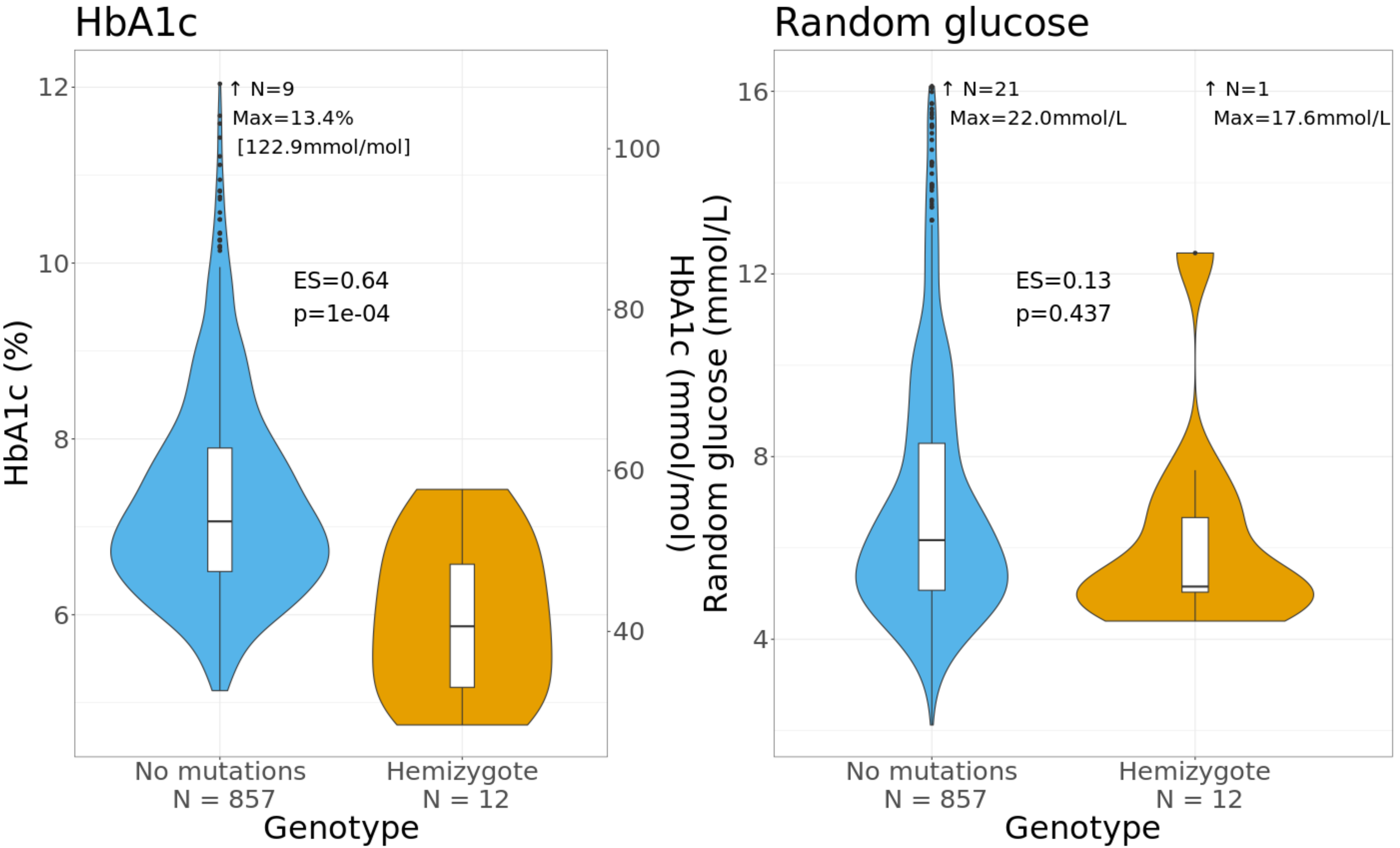
HbA1c
HbA1c (%)
12
10
8
6
↑ N=9
Max=13.4%
[122.9mmol/mol]
ES=0.64
p=1e-04
100
80
60
40
HbA1c (mmol/mol)
No mutations
N = 857
Hemizygote
N = 12
Genotype
Random glucose
Random glucose (mmol/L)
16
12
8
4
↑ N=21
Max=22.0mmol/L
↑ N=1
Max=17.6mmol/L
ES=0.13
p=0.437
No mutations
N = 857
Hemizygote
N = 12
Genotype

Figure S2c.

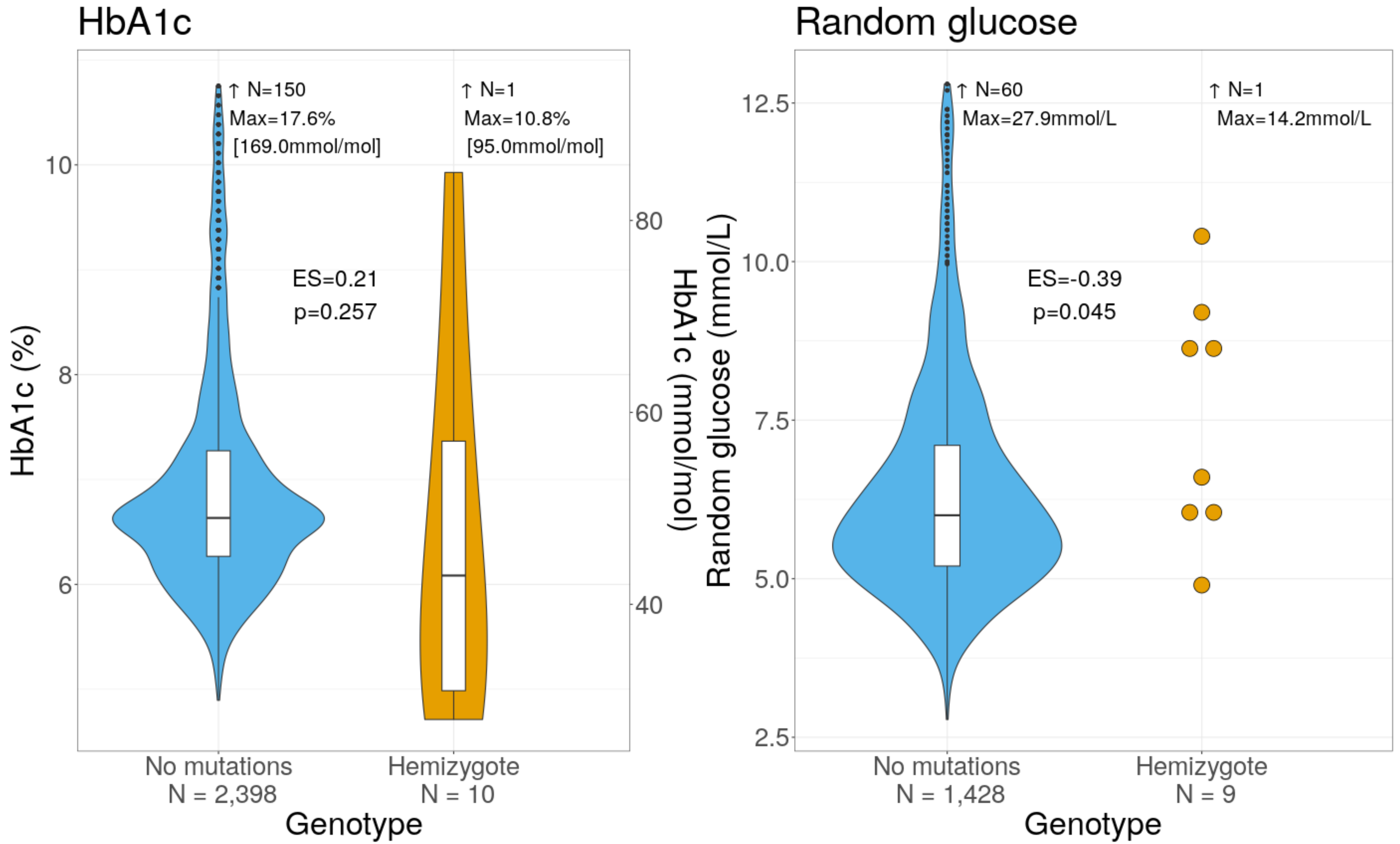
HbA1c
↑ N=150
Max=17.6%
[169.0mmol/mol]
↑ N=1
Max=10.8%
[95.0mmol/mol]
ES=0.21
p=0.257
HbA1c (%)
HbA1c (mmol/mol)
10
8
6
80
60
40
No mutations
N = 2,398
Hemizygote
N = 10
Genotype
Random glucose
↑ N=60
Max=27.9mmol/L
↑ N=1
Max=14.2mmol/L
ES=-0.39
p=0.045
Random glucose (mmol/L)
12.5
10.0
7.5
5.0
2.5
No mutations
N = 1,428
Hemizygote
N = 9
Genotype

Figure S3a.

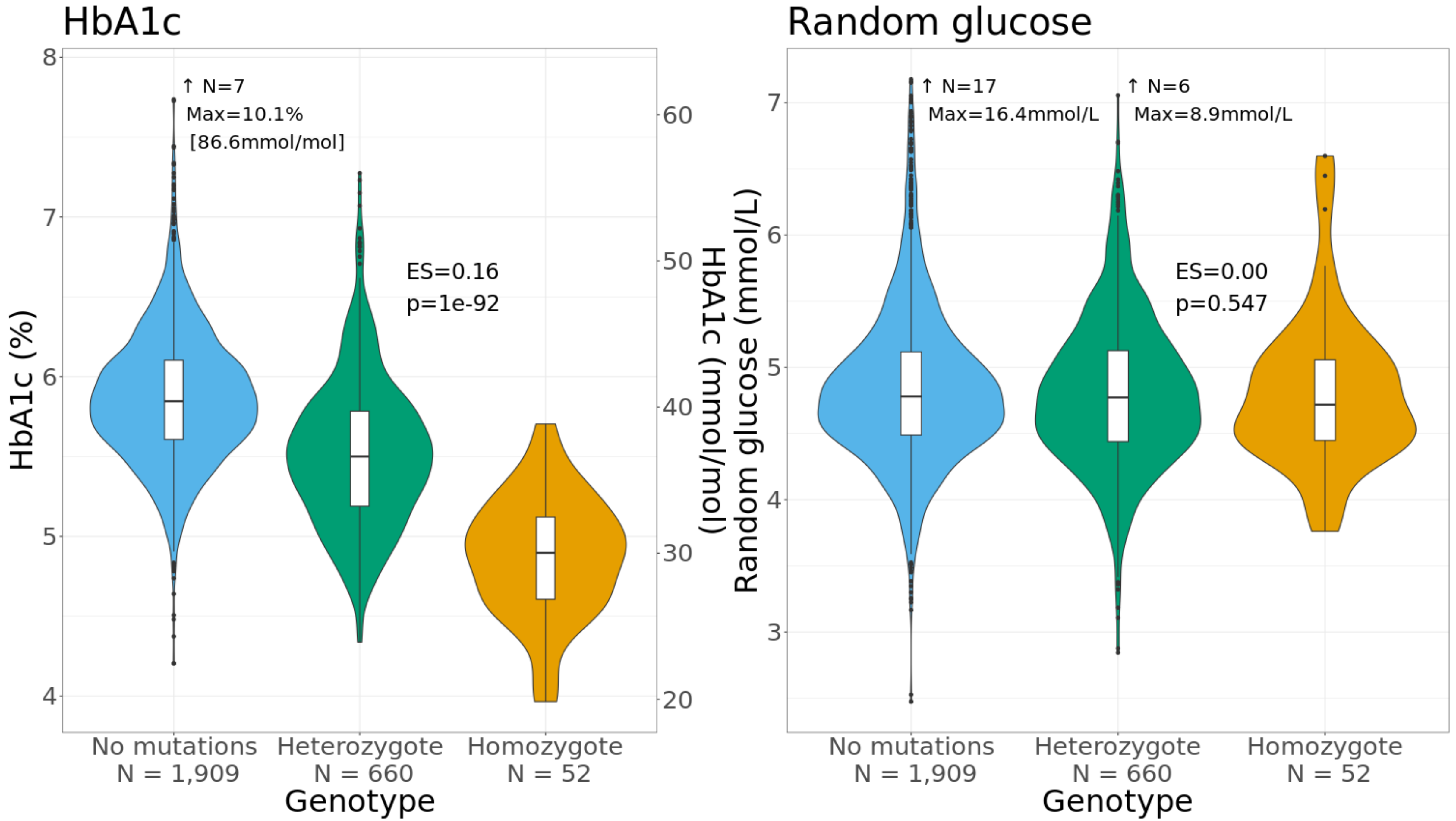
HbA1c
Random glucose
↑ N=7
Max=10.1%
[86.6mmol/mol]
ES=0.16
p=1e-92
HbA1c (%)
HbA1c (mmol/mol)
↑ N=17
Max=16.4mmol/L
↑ N=6
Max=8.9mmol/L
ES=0.00
p=0.547
Random glucose (mmol/L)
No mutations
N = 1,909
Heterozygote
N = 660
Homozygote
N = 52
Genotype

Figure S3b.

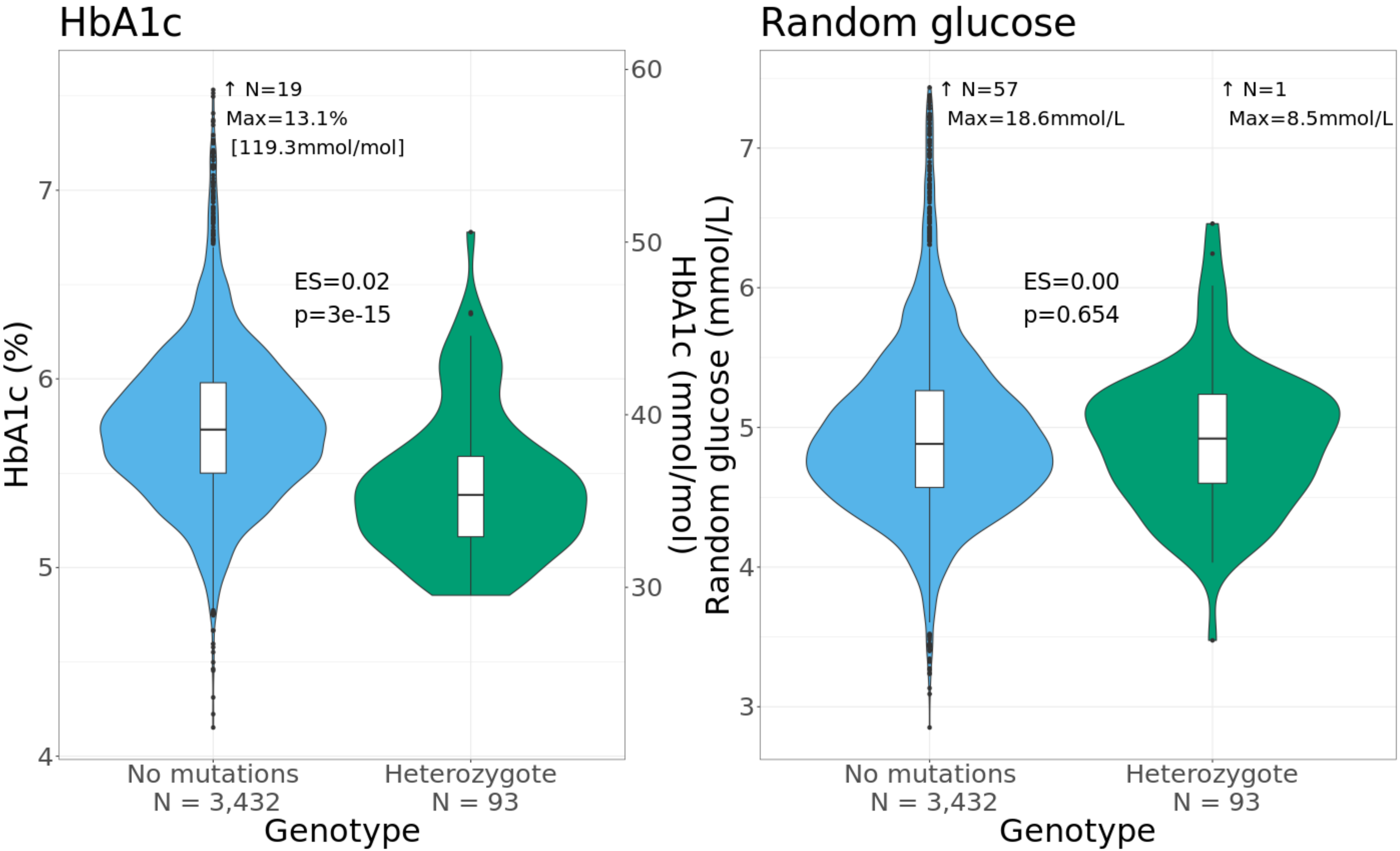
HbA1c
Random glucose
↑ N=19
Max=13.1%
[119.3mmol/mol]
ES=0.02
p=3e-15
HbA1c (%)
HbA1c (mmol/mol)
7
6
5
4
60
50
40
30
No mutations
N = 3,432
Heterozygote
N = 93
Genotype
↑ N=57
Max=18.6mmol/L
↑ N=1
Max=8.5mmol/L
ES=0.00
p=0.654
Random glucose (mmol/L)
7
6
5
4
3
No mutations
N = 3,432
Heterozygote
N = 93
Genotype

Figure S3c.

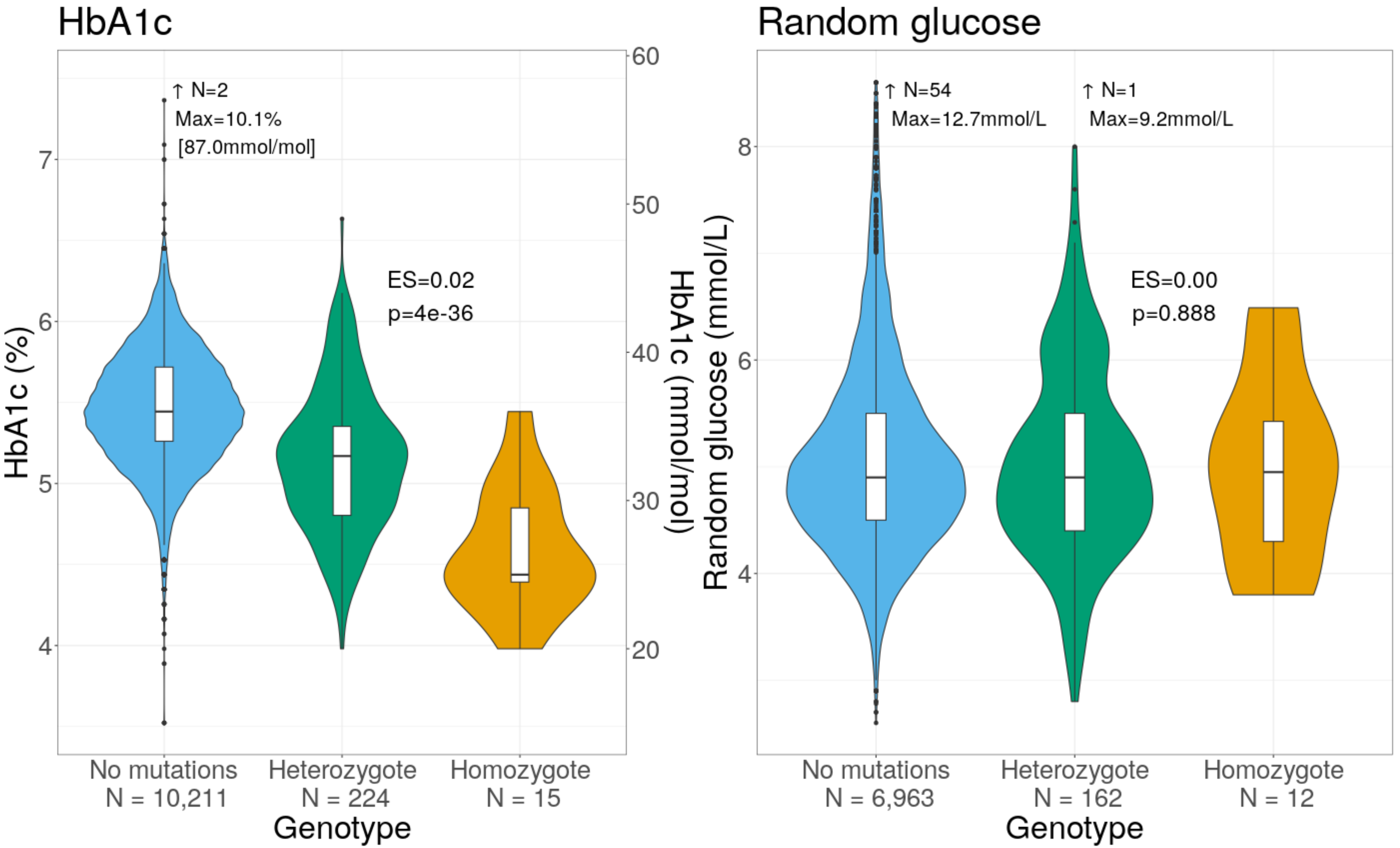
HbA1c
↑ N=2
Max=10.1%
[87.0mmol/mol]
ES=0.02
p=4e-36
HbA1c (%)
HbA1c (mmol/mol)
7
6
5
4
60
50
40
30
20
No mutations
N = 10,211
Heterozygote
N = 224
Homozygote
N = 15
Genotype
Random glucose
↑ N=54
Max=12.7mmol/L
↑ N=1
Max=9.2mmol/L
ES=0.00
p=0.888
Random glucose (mmol/L)
8
6
4
No mutations
N = 6,963
Heterozygote
N = 162
Homozygote
N = 12
Genotype

Figure S4a.

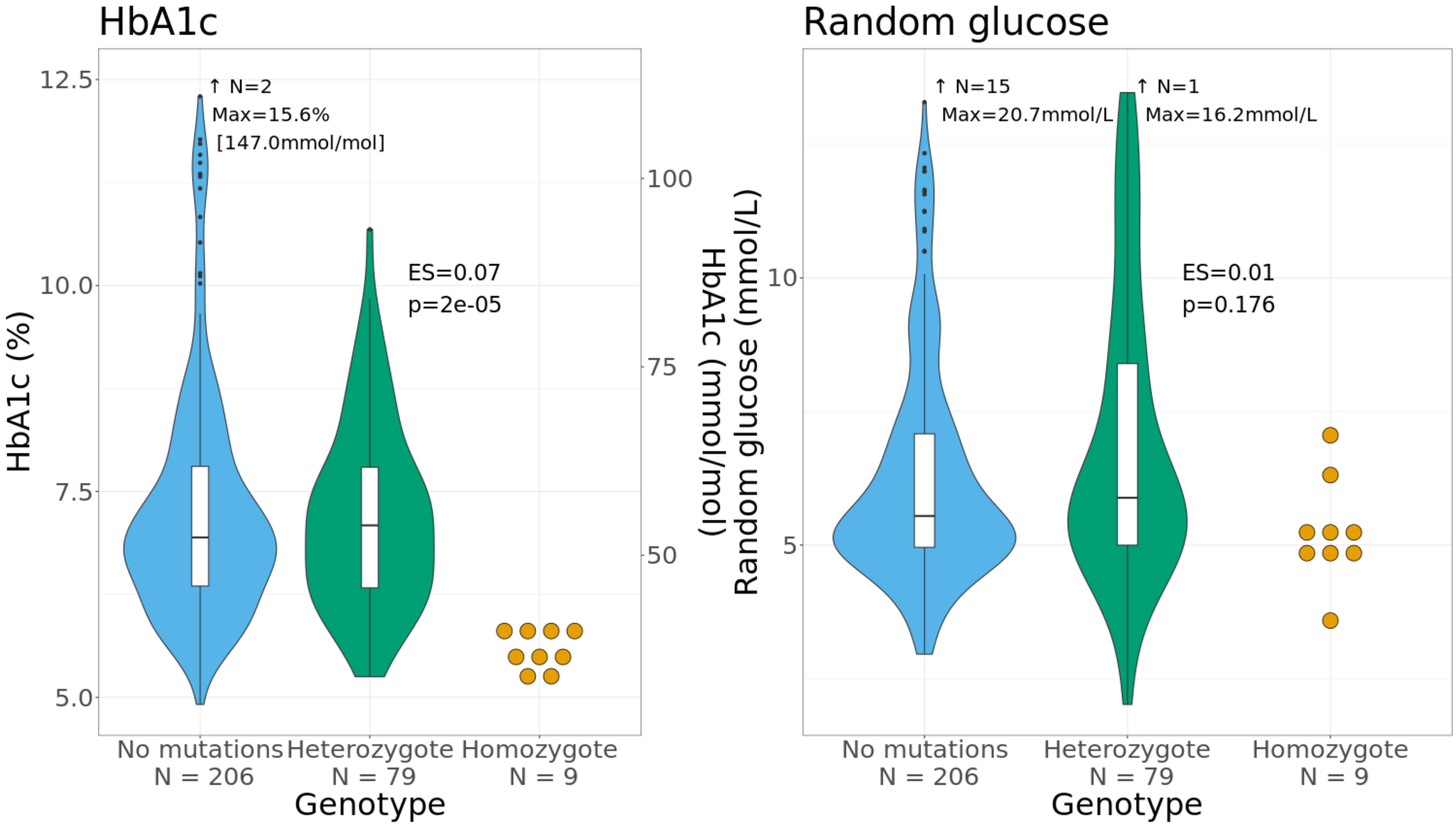
HbA1c
↑ N=2
Max=15.6%
[147.0mmol/mol]
ES=0.07
p=2e-05
12.5
10.0
7.5
5.0
100
75
50
HbA1c (%)
HbA1c (mmol/mol)
No mutations
N = 206
Heterozygote
N = 79
Homozygote
N = 9
Genotype
Random glucose
↑ N=15
Max=20.7mmol/L
↑ N=1
Max=16.2mmol/L
ES=0.01
p=0.176
10
5
Random glucose (mmol/L)
No mutations
N = 206
Heterozygote
N = 79
Homozygote
N = 9
Genotype

Figure S4b.

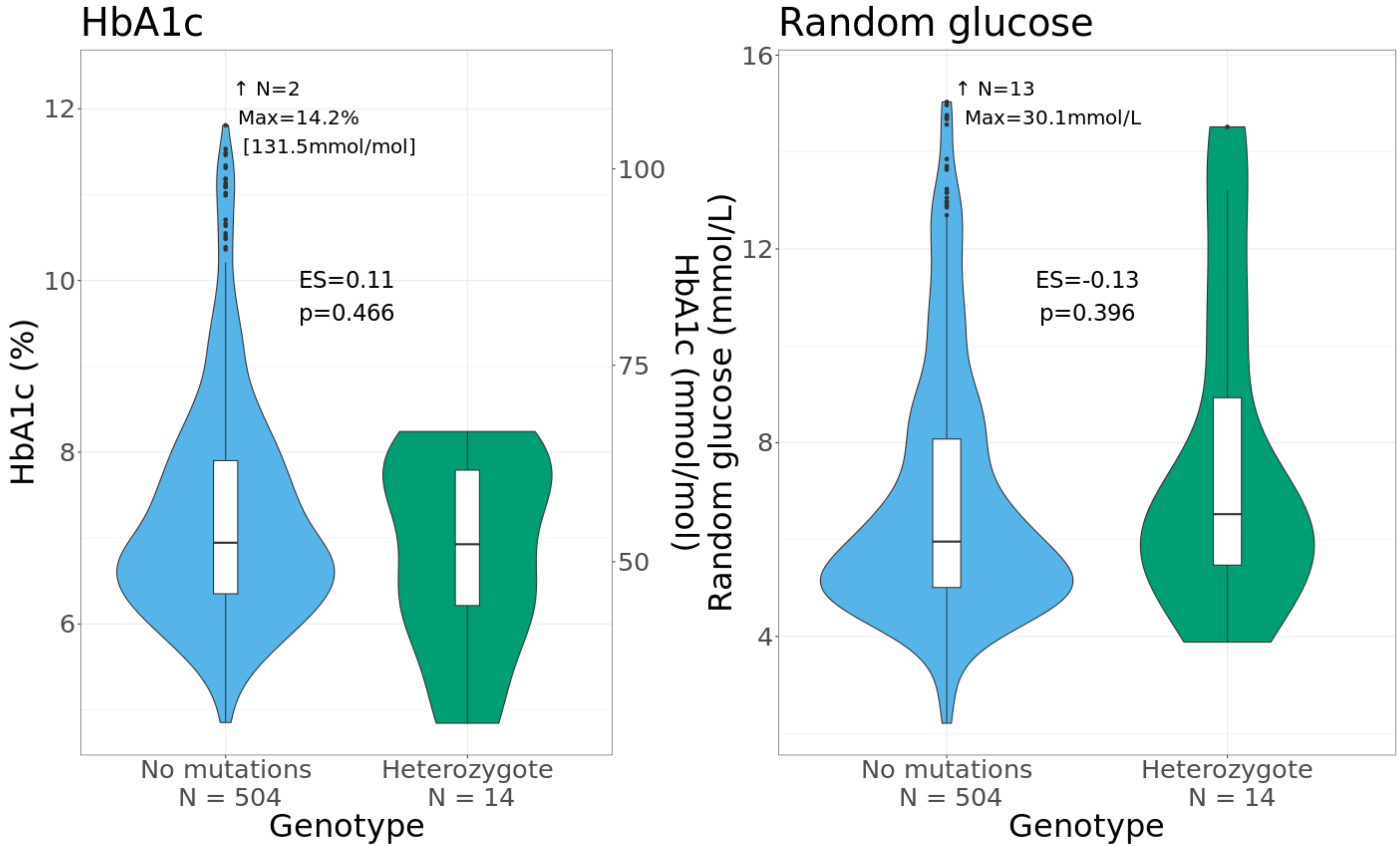
HbA1c
↑ N=2
Max=14.2%
[131.5mmol/mol]
ES=0.11
p=0.466
12
10
8
6
100
75
50
HbA1c (%)
HbA1c (mmol/mol)
No mutations
N = 504
Heterozygote
N = 14
Genotype
Random glucose
↑ N=13
Max=30.1mmol/L
ES=-0.13
p=0.396
16
12
8
4
Random glucose (mmol/L)
No mutations
N = 504
Heterozygote
N = 14
Genotype

Figure S4c.

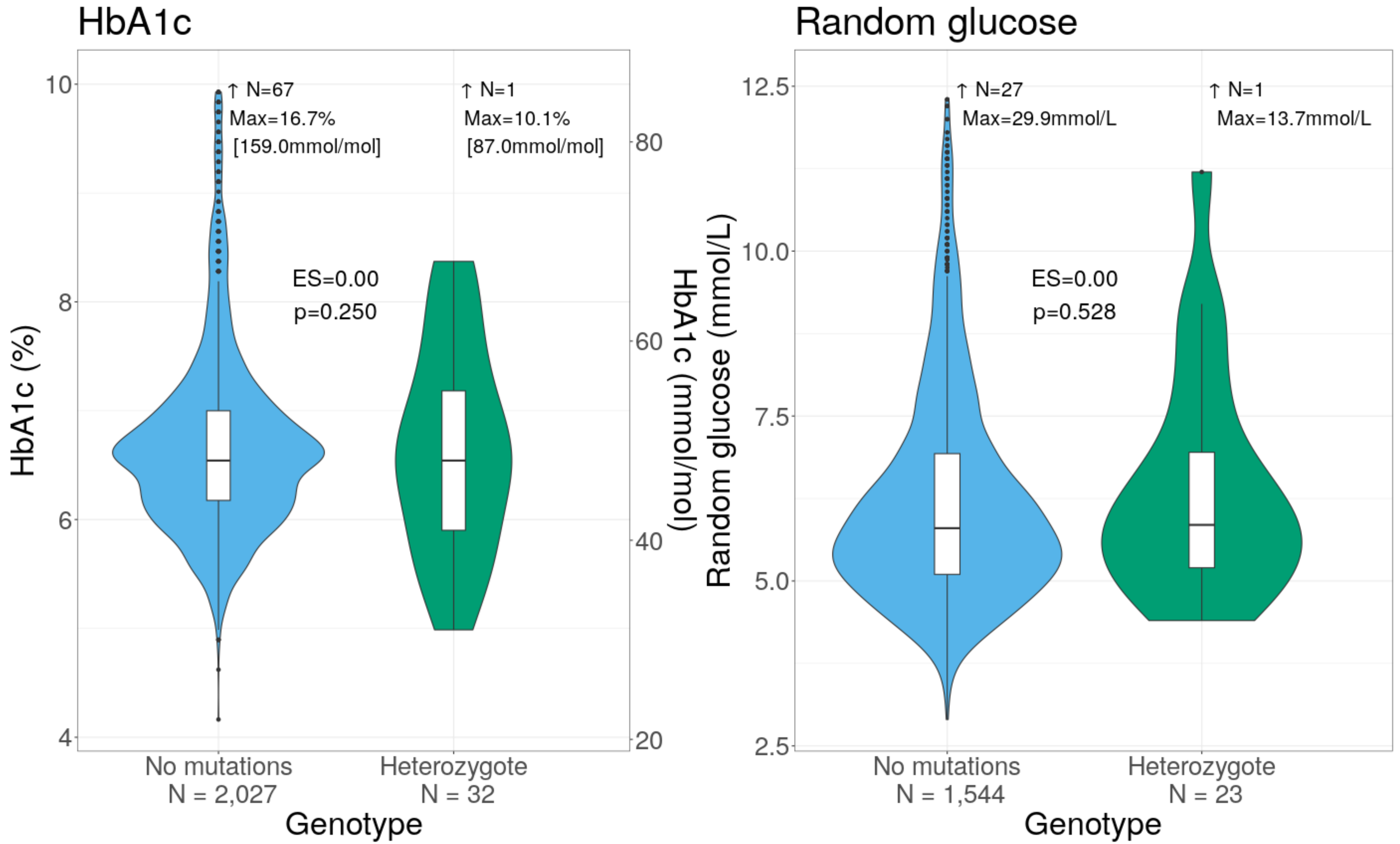
HbA1c
↑ N=67
Max=16.7%
[159.0mmol/mol]
↑ N=1
Max=10.1%
[87.0mmol/mol]
ES=0.00
p=0.250
HbA1c (%)
HbA1c (mmol/mol)
10
8
6
4
80
60
40
20
No mutations
N = 2,027
Heterozygote
N = 32
Genotype
Random glucose
↑ N=27
Max=29.9mmol/L
↑ N=1
Max=13.7mmol/L
ES=0.00
p=0.528
Random glucose (mmol/L)
12.5
10.0
7.5
5.0
2.5
No mutations
N = 1,544
Heterozygote
N = 23
Genotype

Figure S5a.

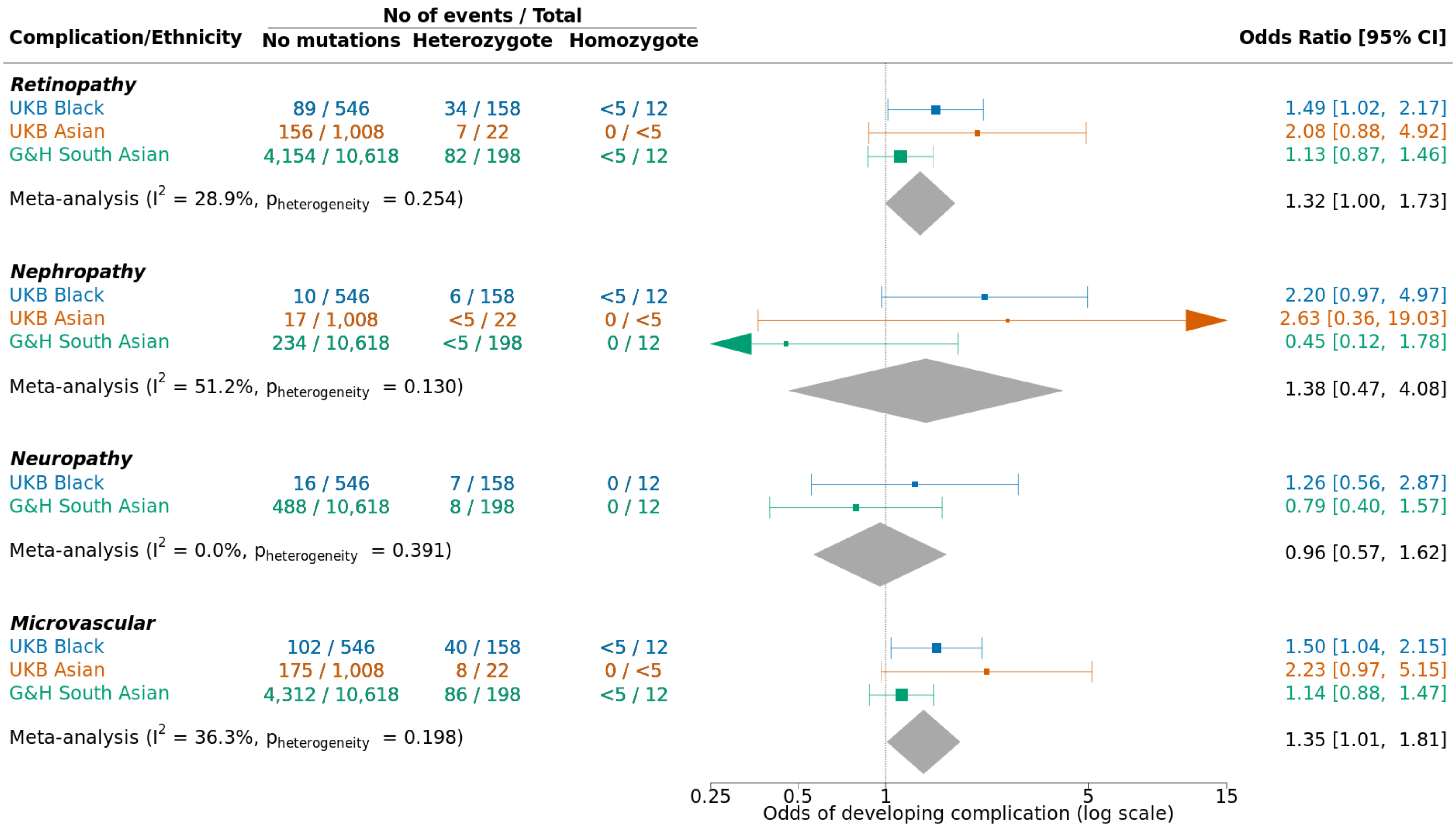

Complication/Ethnicity
No of events / Total
No mutations
Heterozygote
Homozygote
Odds Ratio [95% CI]
Retinopathy
UKB Black 89 / 546 34 / 158 <5 / 12 1.49 [1.02, 2.17]
UKB Asian 156 / 1,008 7 / 22 0 / <5 2.08 [0.88, 4.92]
G&H South Asian 4,154 / 10,618 82 / 198 <5 / 12 1.13 [0.87, 1.46]
Meta-analysis (I^2 = 28.9%, $p_{heterogeneity}$ = 0.254) 1.32 [1.00, 1.73]
Nephropathy
UKB Black 10 / 546 6 / 158 <5 / 12 2.20 [0.97, 4.97]
UKB Asian 17 / 1,008 <5 / 22 0 / <5 2.63 [0.36, 19.03]
G&H South Asian 234 / 10,618 <5 / 198 0 / 12 0.45 [0.12, 1.78]
Meta-analysis (I^2 = 51.2%, $p_{heterogeneity}$ = 0.130) 1.38 [0.47, 4.08]
Neuropathy
UKB Black 16 / 546 7 / 158 0 / 12 1.26 [0.56, 2.87]
G&H South Asian 488 / 10,618 8 / 198 0 / 12 0.79 [0.40, 1.57]
Meta-analysis (I^2 = 0.0%, $p_{heterogeneity}$ = 0.391) 0.96 [0.57, 1.62]
Microvascular
UKB Black 102 / 546 40 / 158 <5 / 12 1.50 [1.04, 2.15]
UKB Asian 175 / 1,008 8 / 22 0 / <5 2.23 [0.97, 5.15]
G&H South Asian 4,312 / 10,618 86 / 198 <5 / 12 1.14 [0.88, 1.47]
Meta-analysis (I^2 = 36.3%, $p_{heterogeneity}$ = 0.198) 1.35 [1.01, 1.81]
0.25
0.5
1
5
15
Odds of developing complication (log scale)

Figure S5b.

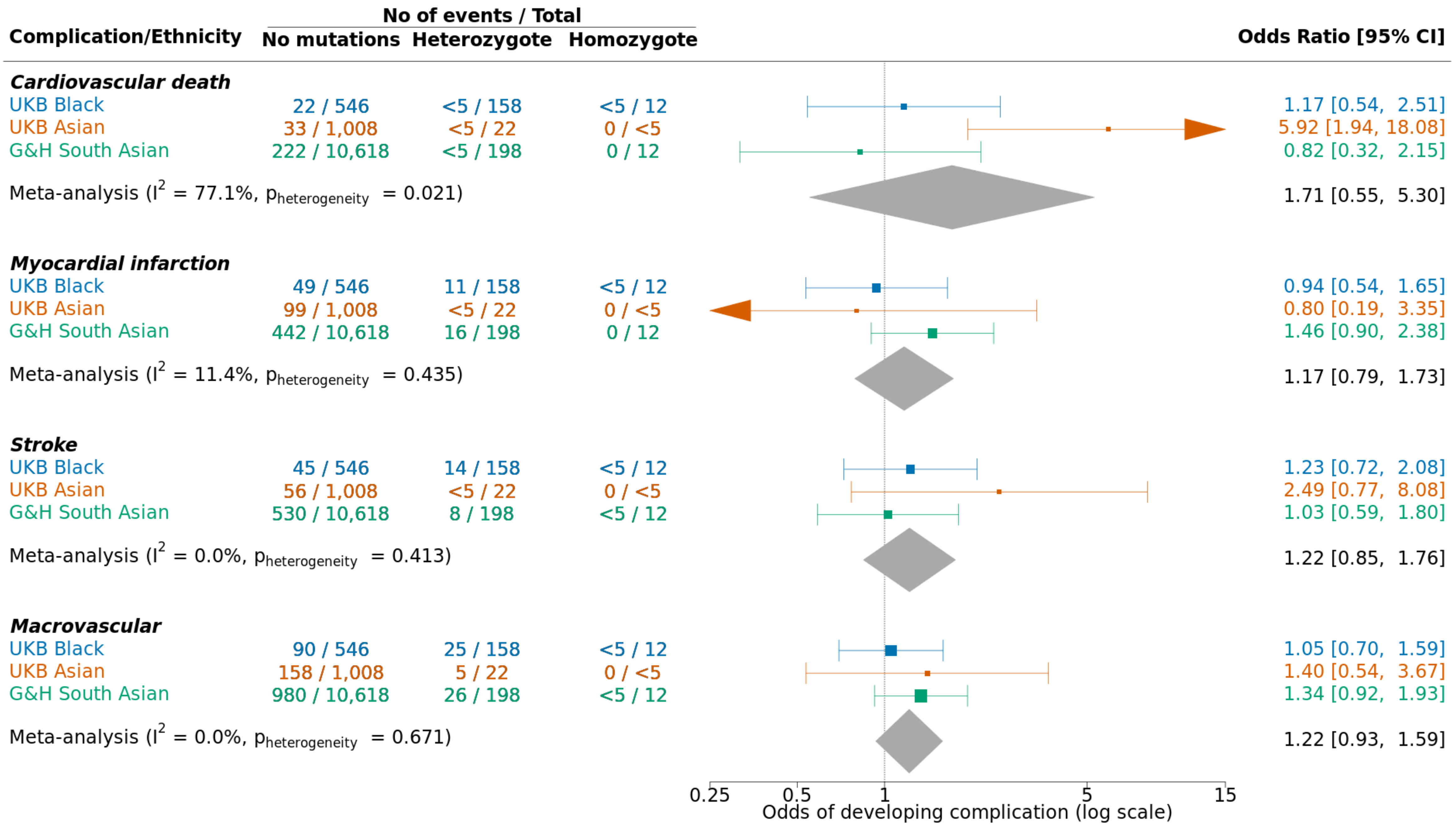
No of events / Total
Complication/Ethnicity
No mutations
Heterozygote
Homozygote
Odds Ratio [95% CI]
Cardiovascular death
UKB Black 22 / 546 <5 / 158 <5 / 12 1.17 [0.54, 2.51]
UKB Asian 33 / 1,008 <5 / 22 0 / <5 5.92 [1.94, 18.08]
G&H South Asian 222 / 10,618 <5 / 198 0 / 12 0.82 [0.32, 2.15]
Meta-analysis (I^2 = 77.1%, $p_{heterogeneity}$ = 0.021) 1.71 [0.55, 5.30]
Myocardial infarction
UKB Black 49 / 546 11 / 158 <5 / 12 0.94 [0.54, 1.65]
UKB Asian 99 / 1,008 <5 / 22 0 / <5 0.80 [0.19, 3.35]
G&H South Asian 442 / 10,618 16 / 198 0 / 12 1.46 [0.90, 2.38]
Meta-analysis (I^2 = 11.4%, $p_{heterogeneity}$ = 0.435) 1.17 [0.79, 1.73]
Stroke
UKB Black 45 / 546 14 / 158 <5 / 12 1.23 [0.72, 2.08]
UKB Asian 56 / 1,008 <5 / 22 0 / <5 2.49 [0.77, 8.08]
G&H South Asian 530 / 10,618 8 / 198 <5 / 12 1.03 [0.59, 1.80]
Meta-analysis (I^2 = 0.0%, $p_{heterogeneity}$ = 0.413) 1.22 [0.85, 1.76]
Macrovascular
UKB Black 90 / 546 25 / 158 <5 / 12 1.05 [0.70, 1.59]
UKB Asian 158 / 1,008 5 / 22 0 / <5 1.40 [0.54, 3.67]
G&H South Asian 980 / 10,618 26 / 198 <5 / 12 1.34 [0.92, 1.93]
Meta-analysis (I^2 = 0.0%, $p_{heterogeneity}$ = 0.671) 1.22 [0.93, 1.59]
0.25
0.5
1
5
15
Odds of developing complication (log scale)

Figure S5c.

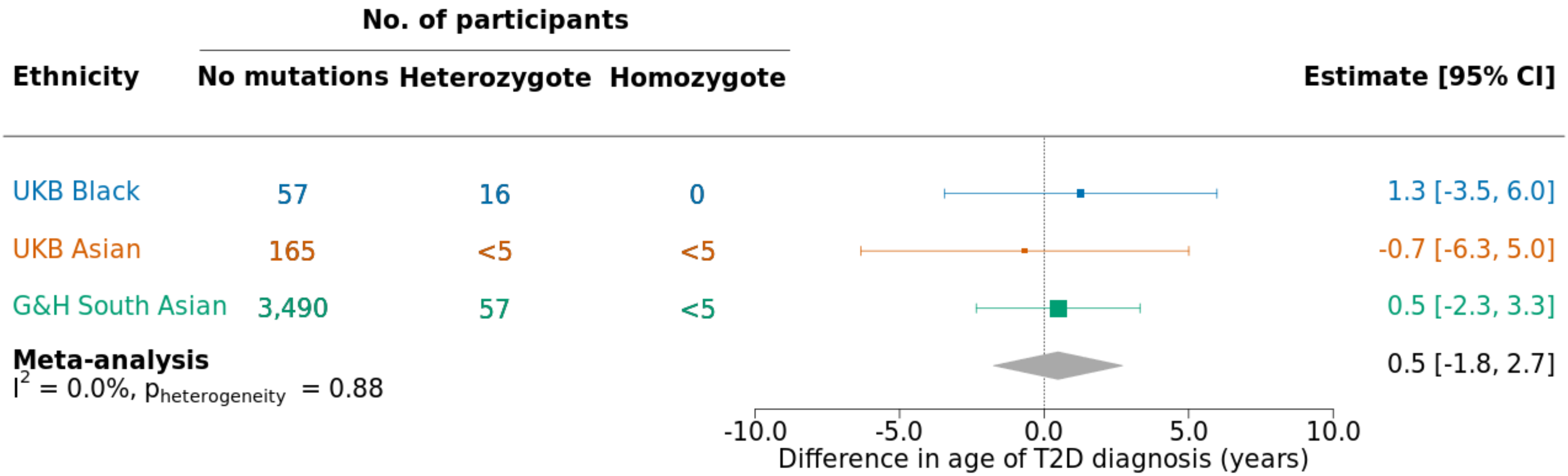
No. of participants
Ethnicity
No mutations
Heterozygote
Homozygote
Estimate [95% CI]
UKB Black
57
16
0
1.3 [-3.5, 6.0]
UKB Asian
165
<5
<5
-0.7 [-6.3, 5.0]
G&H South Asian
3,490
57
<5
0.5 [-2.3, 3.3]
Meta-analysis
I^2 = 0.0%, $p_{heterogeneity}$ = 0.88
0.5 [-1.8, 2.7]
-10.0
-5.0
0.0
5.0
10.0
Difference in age of T2D diagnosis (years)

Figure S6a.

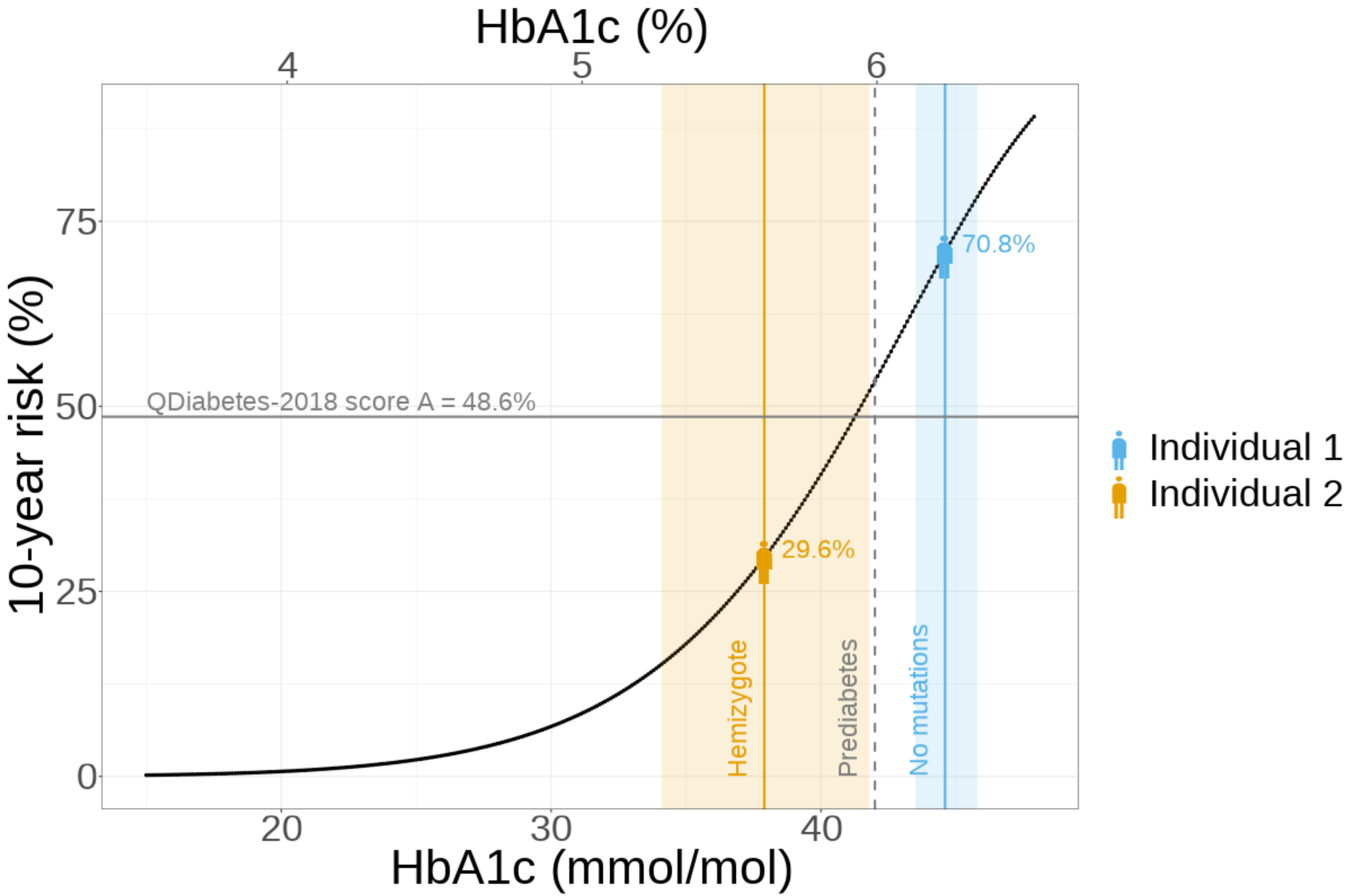
HbA1c (%)
4
5
6
10-year risk (%)
75
50
25
0
QDiabetes-2018 score A = 48.6%
70.8%
29.6%
Hemizygote
Prediabetes
No mutations
20
30
40
HbA1c (mmol/mol)
Individual 1
Individual 2

Figure S6b.

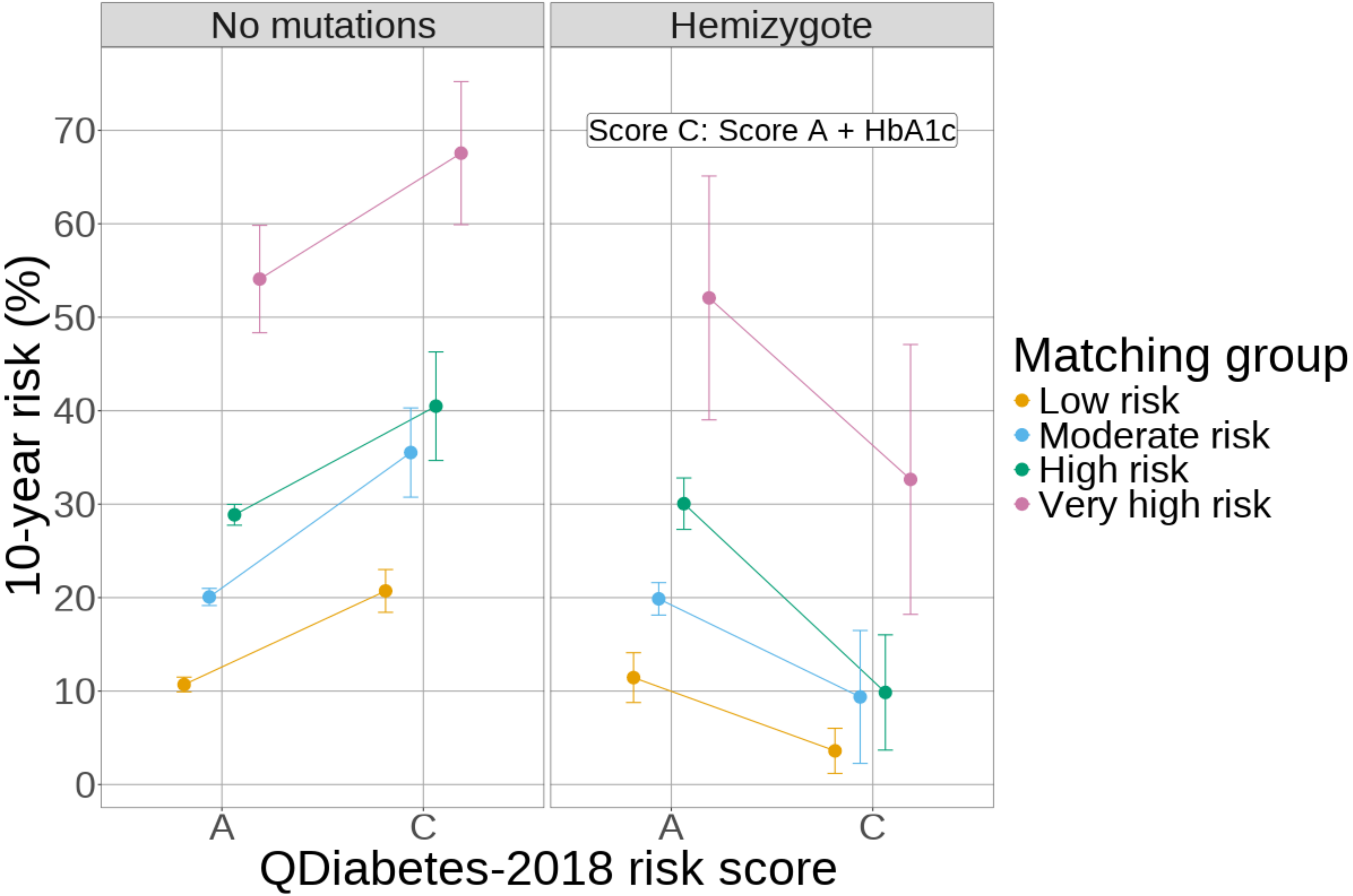
No mutations
Hemizygote
Score C: Score A + HbA1c
10-year risk (%)
0
10
20
30
40
50
60
70
A
C
A
C
QDiabetes-2018 risk score
Matching group
Low risk
Moderate risk
High risk
Very high risk

Figure S7a.

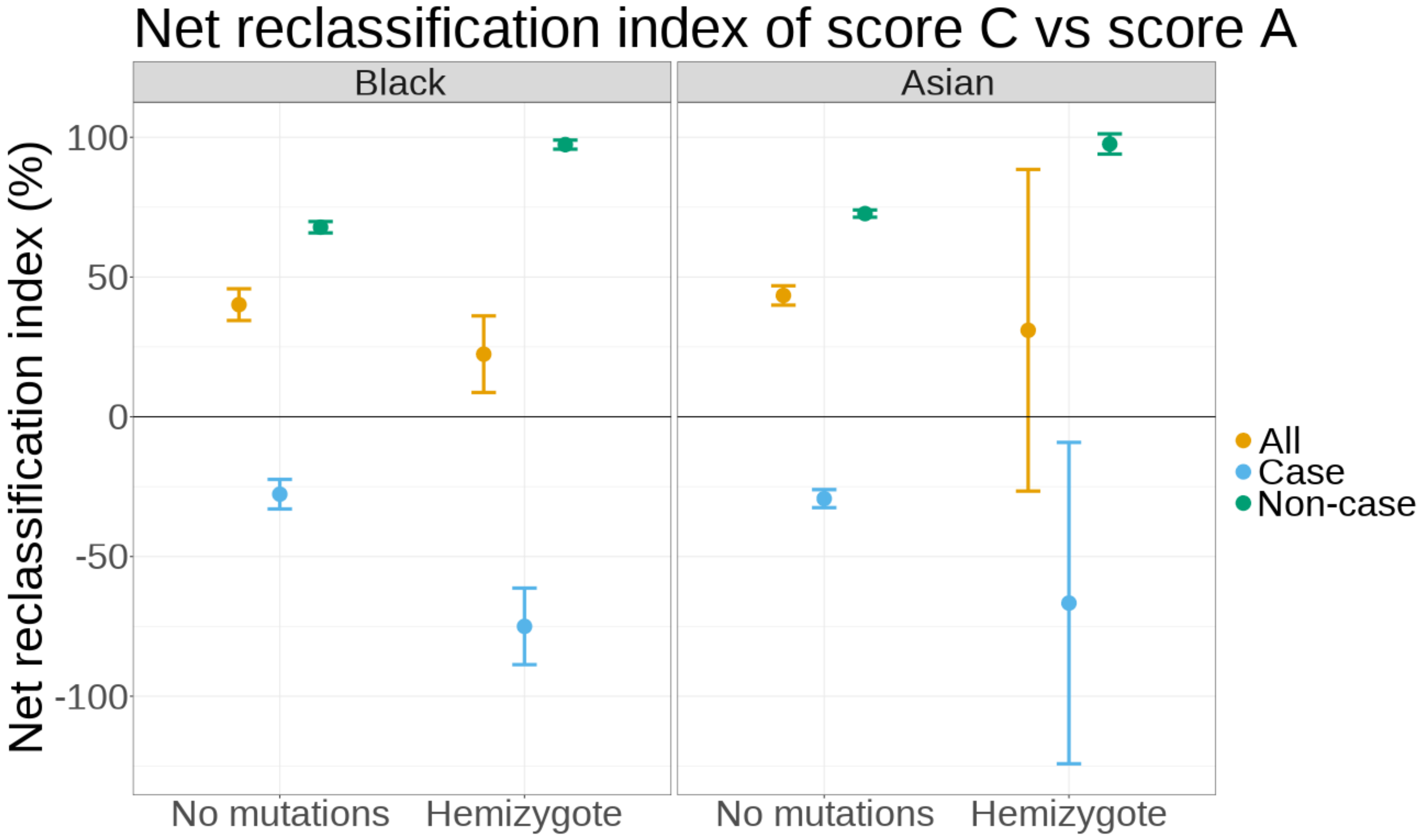

Net reclassification index of score C vs score A
Black
Asian
Net reclassification index (%)
100
50
0
-50
-100
No mutations
Hemizygote
No mutations
Hemizygote
All
Case
Non-case

Figure S7b.

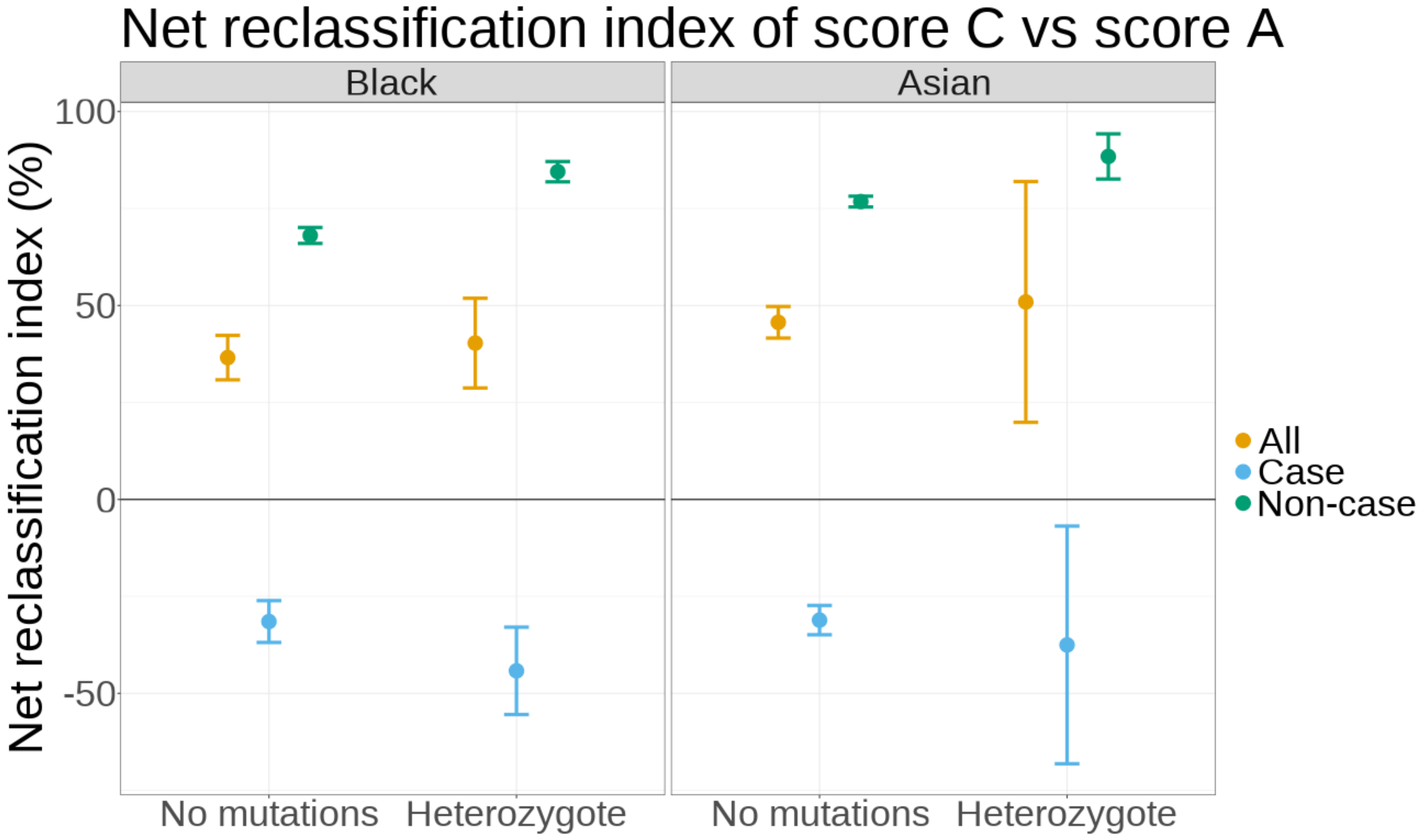
Net reclassification index of score C vs score A
Black
Asian
Net reclassification index (%)
100
50
0
-50
No mutations
Heterozygote
No mutations
Heterozygote
All
Case
Non-case
